# Turkish yoga consumers’ motivation: A scale development study

**DOI:** 10.1371/journal.pone.0359209

**Published:** 2026-10-09

**Authors:** Yeser Eroglu Eskicioglu, Dilsad Coknaz, Sureyya Karsu, Meltem Nurtanıs Velioglu

**Affiliations:** 1 Faculty of Sports Sciences, Duzce University, Duzce, Turkey; 2 Faculty of Sports Sciences, Bolu Izzet Baysal University, Bolu, Turkey; 3 Faculty of Economics and Administrative Sciences, Bolu Izzet Baysal University, Bolu, Turkey; 4 Istanbul Aydin University, Istanbul, Turkey; Sri Ramachandra Institute of Higher Education and Research (Deemed to be University), INDIA

## Abstract

In the growing yoga market, the purchasing behavior of yoga consumers constitutes one of the important research areas. Turkey is a new market for yoga. Information on the reasons and motivations of consumers in this market for purchase of yoga-related services/products is quite limited. For this reason, this study aims to develop a valid and reliable scale to determine the motivations of yoga consumers in Turkey to participate in yoga. The study was structured based on the Scale Development Process. A three-stage process consisting of Item Development, Scale Development and Scale Evaluation was carried out. As a result of the study, a scale consisting of 5 dimensions and 24 items was obtained. The scale shows that yoga consumers in Turkey have intrinsic motivations related to escape and achievement and extrinsic motivations related to visibility, socialization and reference. We hope that the scale developed in this study will contribute to the field in terms of identifying and making sense of the motivations of yoga consumers in similar markets such as Turkey, where yoga is/will be newly developing.

## Introduction

It is derived from the root yoga Yuj, which has a long history of 5000 years before it emerged in India. This ancient Indian practice was first devised by Rishis (sages) in search of happiness and became known as yoga, embodying the concept of unity between body, mind and soul ([1]: 1). Yoga is a contemplative [2,3] and profound, science-based spiritual discipline that integrates both mental and physical practices with the ultimate goal of attaining Paramatman (pure consciousness) and focuses on achieving harmony between the mind and body [4,5]. It is stated that the West first heard of yoga of Eastern origin in 1893, at the “1893 World Parliament of Religions” held in the USA, through the speeches of Indian delegate Swami Vivekananda [6,7]. Vivekananda defined yoga as the “science of the mind,” translated yoga texts from Sanskrit to English [4] and aimed to bring the spirituality of the East, including yoga, to the West [8]. Yoga, which became widespread in the USA in the 1960s [9]:2), continues to spread all over the world with the influence of globalization [10–13]. Data from 2024 show that there are 300 million yoga participants in the world and the yoga industry has reached a value of 338 billion dollars (Wellness [14], and the increasing market share of yoga [15,16] is remarkable.

Yoga, which has been preserved and encouraged by yoga masters – Gurus – since ancient times [5], has the same basic dynamics worldwide, but there are significant differences between the Eastern yoga teachings and the way the West interprets it [17]. For example, while yoga is practiced as a holistic discipline encompassing mental, physical and spiritual aspects in India, it is perceived as a physical practice aimed at relieving pain and stress in the United States and the United Kingdom [18].

Yoga works on the body, mind, emotion and energy levels of a person [19]. Despite this deep philosophy, it is seen that the acceptance of yoga in Western culture is more associated with physical health and fitness [20], and it has been adopted by the fitness world [10,21] and it is adapted to appeal to gym members [22].

Yoga, which is an integral part of the culture and daily life in the East, is perceived as a 60-minute lesson/activity applied to relieve the stress of the day, adapted to a busy lifestyle in the West [17]. It is also stated that yoga is in the process of commercialization, and commodification [23,24] and inextricably linked to consumer culture [25–27] in its Westernized forms [4,10,27]. However, for example, the International Yoga Federation sees yoga as a sport [28]. Since 2016, the institution has been organizing the World Yoga Championships, which include the categories of Yoga acrobatic, Yoga artistic, Yoga asana, Yoga athletic, Yoga dance, Yoga flow, Yoga rhythmic [29]. Yoga activities that maintain global interest, such as the rise in the United States on June 21^st^ International Yoga Day (Wellness [14], are reflections of yoga in its interpretation in Western culture.

Although private studio memberships, expensive clothing, etc. are key elements in the commercialization of yoga, except for very wealthy metropolitan areas, yoga in the East is primarily a tradition practised at home, alone or one-on-one, and is not an activity to be participated in [17]. Wilberg [9]:2) also states that yoga is now seen as a commercial tool for marketing yoga accessories (mats, bands, belts, clothes, and blocks), interactive videos, and personal fitness regimes and that there is no continuity of the ancient traditions.

However, yoga is seen as an export product in its own land, for example, Mukherjee [30] states that exporting yoga can be part of traditional and preventive health exports and that it is valuable for the production and export of yoga accessories such as yoga mats, blocks, clothes, and other equipment.

The evaluation of yoga from very different perspectives, the active study of the commercial value of yoga by academia, businesses, and organizations, the increase in the consumption of yoga, and the growth in the yoga market make it a matter of curiosity about how yoga consumers’ purchasing behaviors of yoga and yoga-related accessories, training, etc. have developed. Understanding why and how consumers decide to purchase a product is the driving force behind the marketing process [31].

Internal, external and situational factors shown in Fig 1 are effective in the consumer’s purchasing process [32].

**Fig 1:**
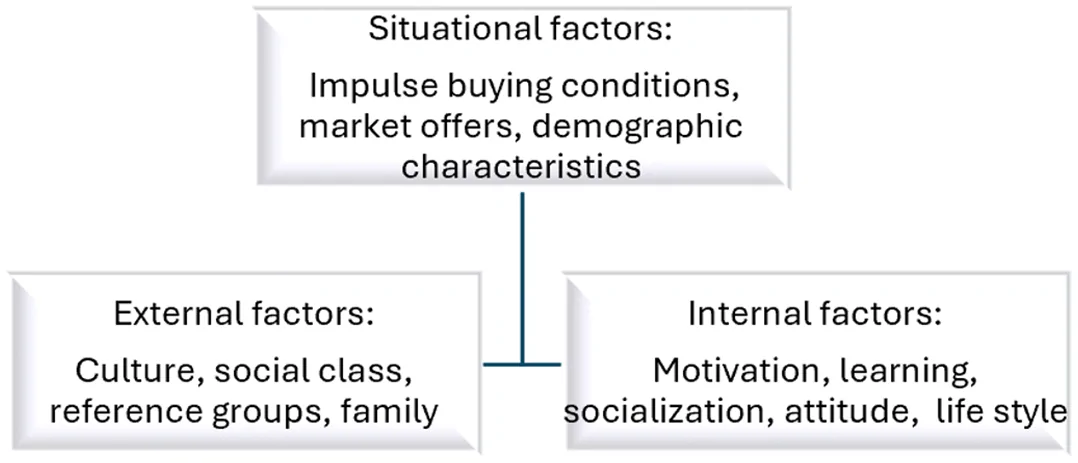
Factors Affecting Purchase.

Motivation, which is an effective internal factor in purchasing, is used to explain the reasons for individuals’ behaviors, and *what initiates and directs* the behavior [33,34]. Motivation, derived from the Latin word “movere”, means “to take action, to set in motion” [35].

The concept of motivation, which tries to find an answer to the question “why” regarding consumer behavior in marketing, was deemed worthy of examination within the framework of the reasons for yoga participation of yoga consumers (yoga participants), who are the focus of our study, on the basis of the following:

(i) Quantitative studies on yoga motivations have little empirical data on why people adopt yoga practice [20,36],
(ii) Existing studies are often based on focus groups and small samples [20],
(iii) The importance of understanding the motivation and satisfaction of yoga consumers for leisure service providers and researchers in the context of its increasing popularity [37],
(iv) The fact that yoga participants, especially in the West, remain a limited market, with female consumers in terms of gender [36,38–42] and affluent consumers in terms of income group [41–43],
(v) Emphasis made by Cagas et al. [39] that there is a need to elucidate the factors affecting the purchase of yoga since an understanding of participation motivations can help differentiate promotional messages for different population subgroups,
(vi) Research on yoga is mostly structured in markets where yoga has been present for a long time, and studies on consumer purchase motivations are limited in markets where the yoga experience has just started to spread, such as the Turkish market, etc.

### Yoga motivation sources

According to Brems et al. [44], the most common motivation sources in yoga are the desire to exercise, increased flexibility, better posture and balance, relaxation, emotional balance, and improving one’s health. Penman et al. [45] found that yoga participants in Australia generally started doing yoga for health and fitness purposes and continued to do yoga for stress management, and Quilty et al. [46] similarly found that participants in Texas did yoga for general health, physical exercise, stress management, and to alleviate a health problem. Among the reasons for yoga participants in the United States, flexibility ranked first, followed by stress reduction, fitness, health improvement, strength, mental health, spiritual development, weight loss, physical therapy, and health during pregnancy [47]. While the initial motivations of yoga participants in Germany were relaxation and protection (prevention), their continuing motivations were established as prevention and then spirituality [48]. Data from China show that yoga participants are motivated by improving their physical condition, improving psychological condition and appearance, establishing social connections and creating social isolation [37]. Cartwright et al. [36] reported that the motivations of yoga participants in the United Kingdom were initially general health, fitness and flexibility, while some of those who started yoga due to a physical or mental health problem focused on spirituality, stress management and social interaction over time. Cagas et al. [39] found that positive affect, health-fitness and agility motivations were generally prominent in participants of different types of yoga, but compared to exercisers, postural yogis and yogis were more motivated by spirituality, mind-body integration, coping and stress management, and that some people were interested in yoga for its health and fitness benefits alone, while others saw yoga as more than just physical exercise.

In Turkey, where this study was conducted, academic studies on yoga began in 2015 [49], and academic studies addressing the motivations of yoga participants are generally qualitative studies. The results of qualitative studies conducted with small samples show that yoga participants in Turkey have motivations such as health, reference sources, curiosity, economic reasons, aesthetics and having fun, health spirituality and professionalization [50]; psychological relaxation, curiosity/giving it a try, professional expectation/contribution, spiritual/spiritual, calming the mind, physical exercise/physical good appearance, body health [51]; financial concerns, physical and mental health, the contraction in leisure activities during the pandemic [52]; healthy life, creating awareness, reducing pain, staying calm, coping with stress and increasing positive thinking [49].

Although it is a relatively new field of practice [53], the consensus of this research team is that yoga is becoming increasingly widespread in Turkey based on observations such as the opening of many yoga centers, the increase in advertisements of yoga centers on social media, celebrities sharing their yoga practices on social media, and the increasing preference of some individuals to quit their current professions and become yoga instructors.

Yoga activities in Turkey were institutionalized with the organization of the Yoga Federation in 2013 [54]. It is thought that there has been a rapid increase in the number of yoga participants and yoga centers [55]. It is also a fact that the number of people who do yoga in Turkey has not yet been determined exactly [49,56], as in other studies reporting that they could not access numerical data on yoga participants, yoga instructors, and yoga training centers [36,45]. In this context, although it is known that there has been an increasing interest in yoga participation in Turkey over time, it is seen that the behavior of yoga consumers has not been investigated much.

In the literature review, it was seen that exercise, sports or physical activity-based scales such as Exercise Motivation Scale-2 [39], Physical Activity and Leisure Motivation Scale [37] and Sport Motivation [57] were used in the measurement of yoga motivations. It was seen that the studies conducted using a scale were conducted with yoga participants in developed markets where yoga had been practised for a long time [58–60] or in Eastern countries. Undoubtedly, the motivations and responses of consumers in markets where yoga has been experienced for a long time may differ from those of consumers in markets where it has just begun to be experienced [46]. In addition, consumers’ purchasing motivations are subjective [61]. Different groups may have different perceptions about the importance of consumer motivation dimensions [62]. In this context, yoga motivations in countries that have been practicing yoga for a longer time may differ from Turkey.

In the enquiries conducted, no scale developed for yoga motivations in Turkey was found. It was determined that the scale studies that could be reached were scales for behavioral elements other than motivation, such as the yoga self-efficacy scale adapted by Tetik Küçükelçi [63] from Birdee et al. [64]; and the yoga belief scale adapted by Tetik Küçükelçi [65] from Sohl et al. [66].

Based on this literature and in the light of the information, the aim of our research is to develop a scale to determine the motivations of yoga consumers (yoga participants) to participate in yoga in Turkey, where yoga has just begun to develop.

## Method

In the scale development process, DeVellis [67] and Boateng et al. [68] were examined, and the 3-stage methodological process shown in Fig 2 was followed.

**Fig 2:**
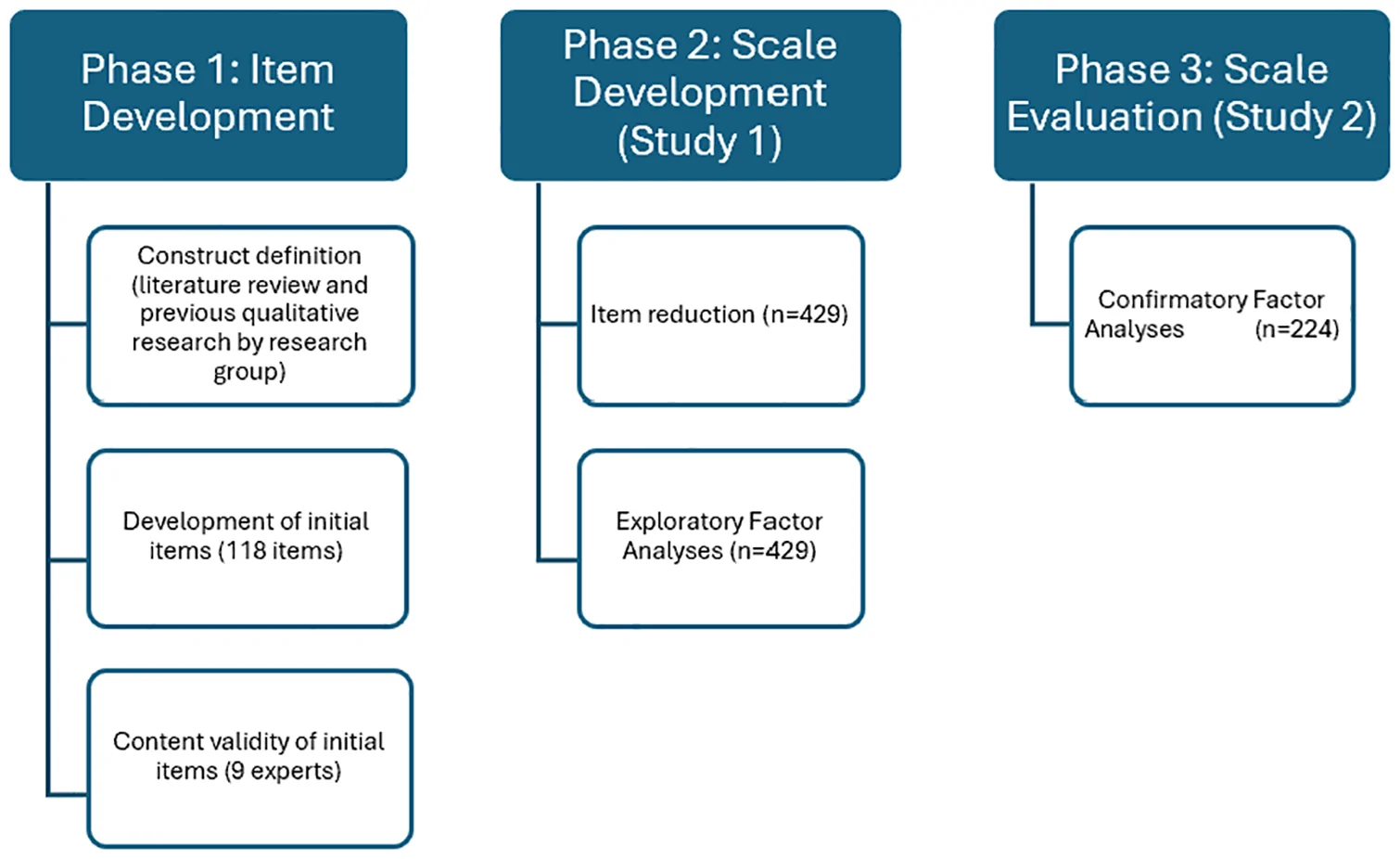
Methodological Process Followed for the Development of the Scale.

### Phase 1. Item development

At this stage, the determination of the items to be included in the scale and the content validity procedures for the items [68] were carried out.

***Construct Definition:*** The creation of the theoretical structure of the scale was guided by the qualitative research conducted by the same research group in 2018 to determine the perception and motivation of yoga consumers in Turkey towards yoga [50]. In this research conducted in 2018, the initial motivations were health, reference sources, curiosity, economy, aesthetics and having fun, while the continuation motivations were health, spirituality, economic orientations, professionalization, promotional activities and the atmosphere of the place. In addition, studies on yoga motivations have also been examined [20,36,37,43–46,57,69–72]. In the context of the qualitative research conducted in 2018 and other studies reviewed, the structural elements of yoga consumers’ motivations were shaped around internal elements such as spirituality, escape, aesthetics, health and achievement, and external elements such as fashion, visibility, reference source, socialization, economy and physical activity/exercise.

***Development of Initial Items:*** As a result of the enquiries, an initial item pool consisting of 118 items was created based on the theoretical basis. In creating the initial item pool, the items to be included in the scale were discussed and prepared by the researchers, taking into account the suggestions made by DeVellis (2017: 73–85), short sentences were used in the item writing, the number of items was increased by writing alternative items in order to find the item that would best express the concept, and negatively/reverse scored items were not written. The use of the “Five-Point Likert Type” measurement tool was preferred for the scale, and the statements were rated between 5 and 1 between “strongly agree” and “strongly disagree”.

***Content Validity of Initial Items:*** The draft scale consisting of 118 items was submitted to expert opinions for content validity. It is generally recommended that the number of experts be between 5 and 40 in content validity studies [73–76]. At this stage, opinions were received from a total of 9 experts from the fields of psychology, sports psychology, and sports sciences, some of whom had previously come into contact with yoga. The experts made evaluations based on whether the scale items represented the characteristic to be measured, and whether the items were sufficiently clear. According to Lawshe’s [74] content validity index, the minimum value required for 9 experts in terms of consensus is 0.75. 67 items of the 118-item draft scale were eliminated because they were below 0.75, and 51 items with a value of 0.77–1 on which the experts reached a consensus remained. After adding questions containing demographic information, the scale was presented to a Turkish language expert for review in terms of writing and Turkish language, and the scale items were reviewed to ensure the linguistic clarity of the measurement tool.

### Phase 2: Scale development

Boateng et al. [68] recommend conducting exploratory factor analysis (EFA) within the scope of an appropriate sample size to reveal the factor structure underlying the expressions at this stage. In this direction, the first evaluation of the remaining 51-item scale was made after expert opinions, and the items that did not consistently provide initial reliability and validity were removed, and the optimum number of factors or areas was determined.

***Data Collection and Sample:*** The data for this phase were collected from volunteer participants in the form of an online survey via Google Forms during the Covid-19 pandemic quarantine period between April 15 and July 15, 2021. Data were collected after obtaining ethical approval from the Bolu Abant Izzet Baysal University Human Research Ethics Committee (Protocol No. 2021/92). Participants’ consent was obtained electronically, and participants were asked to select the “I participate in this study voluntarily” option when documenting their consent to participate. This procedure ensured that all participants joined the study voluntarily and with full awareness. Additionally all participants were 18 years of age or older; thus, no parental or guardian consent was required. In order to reach the participants, the managers of yoga centers in Turkey were first contacted via e-mail and informed about the study, and they were asked to share this information with both former and current members. The Google Form was then sent to the members who responded, via e-mail and WhatsApp. The data obtained were stored on a hard drive and were accessible only to the researchers. The first part of the measurement tool is about demographic (age, gender, marital status, income, region of residence, type of yoga, frequency of yoga, duration) information, and the second part is about motivations for participating in yoga. In scale development studies, it is recommended to reach a sample size of 10–15 times [77] or 5–10 times [78] the number of items in the scale. At this stage data was initially collected from 667 individuals using the convenience sampling method. As a result of the examination of missing, incorrect and extreme values, 429 valid data were obtained. The high level of data loss is due to the large number of blank, incorrect and incompletely filled open-ended questions in the demographic information section. Of the 429 participants at this stage, 52.2% live in the provinces in the western region, 94.9% are female and 50.3% are married. 35.2% are between the ages of 26–35, 32.4% are between the ages of 36–45. With 2021 figures, the incomes of the participants are in a wide range between 1000–75,000 TL, and the average income is 6927.35 TL. 17% of the participants have been doing yoga for less than 1 year, 40.6% for 1–3 years, 18.2% for 4–6 years, and 24.2% for more than 7 years. The frequency of yoga participation was 1–2 times a week for 54.8%; 3–4 times for 23.8%; 5–6 times for 12.1%; and every day for 9.3% of the participants. Among the yoga types that the participants answered open-endedly, it was seen that Hatha yoga was the most frequently done type with 52.4%. [79]

***Item reduction:*** Before the EFA process, it was examined whether the data was normally distributed, and it was determined that the Skewness and Kurtosis values (Skewness −1.106–1.352; Kurtosis −1.741–1.477) were between −2 and +2 [80], which are accepted in the literature. By looking at the correlations between the items in the scale, it was checked whether the correlation was.30 and above [81], then in the 51-item draft scale, 18 items with item-total correlations below.30 were removed from the scale, and the EFA process was continued with the remaining 33 items.

***Exploratory Factor Analyses Process:*** In order to determine whether the data is suitable for EFA at this stage, the Kaiser-Meyer-Olkin (KMO) coefficient (<.70) and the Bartlett sphericity test value (χ2 < .05) were calculated (KMO = .871, χ2 = 9801.905; sd = 528, p < 0.01), and it was determined that the values met the criteria values specified in the literature [82]. In scale development studies, factor analysis was used to examine whether there was a certain order between the responses given by the participants to each item in the draft scale [78] and to determine whether the items were separated into a single factor or more than one factor. In factor analysis, factors with an eigenvalue of 1 or greater than 1 are considered important factors [83]. There was no limitation on the number of factors in the scale, and factors with an eigenvalue greater than 1.00 were included in the scale. In the analysis process that continued with the remaining 33 items, the Oblique (Oblimin) rotation method was used to find conceptually independent but empirically related factors with principal axis factor analysis [84]. The criteria for keeping the items were as follows:

(1) The item has a loading of over.40 on its own factor [85]
(2) The difference between the item factor loading values in the two factors is more than 0.10 [86,87];
(3) The item fits the factor conceptually [88].
(4) At least three variables under each factor [89]

After the analyses based on the criteria mentioned above, the overlapping items that were below 0.40 in factor loading and loaded on more than one factor and had a difference of less than 0.10 between them were removed one by one at each stage, and the analyses continued until no overlapping items remained. In the relevant analyses, 6 items (M17, M27, M39, M41, M46, M47) included in the scale were eliminated. In accordance with the criterion of at least three variables under a factor [89], since two variables remained under the relevant factor, items M12 and M13 were also eliminated from the scale. After the items were removed, 25 items remained on the scale. At this stage, it was seen that all items related to spirituality, aesthetics, health, economy, and physical activity/exercise were eliminated from the Yoga Motivation Scale (YMS).

### Phase 3: Scale evaluation

In this phase, CFA and reliability analyses were conducted by following the steps suggested by Boateng et al. [68].

***Data Collection and Sample:*** The data for this phase were collected in the form of an online survey via Google Forms between May and August 2024. After the EFA, the remaining 25 items were renumbered by shuffling the order (1–25) and sent to the participants in this manner. The first part of the measurement tool is about demographic information (age, gender, marital status, income, region of residence, type of yoga practised, frequency of yoga practice, duration), and the second part is about motivations for participating in yoga. As a result of the EFA, data was collected from 270 people by using convenience sampling method to verify the validity of the scale consisting of 5 dimensions and 25 items. As a result of the missing, incorrect and extreme value examination, 224 valid data were obtained. 42% of the 224 participants in this phase live in the provinces in the Northeast, 89.3% are female and 51.3% are married. 35.7% are between the ages of 26–35, 29% are between the ages of 36–45. According to the figures for 2024, 66.5% of the participants have a household income of 42,000 TL and above. 34.8% of the participants at this stage have been practicing yoga for less than a year. Those who have been practicing yoga for 1–3 years are 30.8%, those who have been practicing yoga for 4–6 years are 22.3%, and those who have been practicing yoga for more than 7 years are 12.1%. The vast majority of participants (67%) do yoga 1–2 times a week. Among the yoga types that the participants answered open-endedly, it was seen that Hatha yoga was the most frequently performed type with 44.6%.

***Confirmatory Factor Analysis Process:*** Confirmatory Factor Analysis (CFA) was applied to the 5-factor and 25-item scale obtained after EFA to verify the construct validity. This process was performed on a sample different from EFA. In order to test the pre-CFA analysis assumptions, the corrected item-total correlation values of the items were first examined. Since the item-total correlation of the 5^th^ item was below 0.30 [81], it was removed from the scale, and the CFA process was carried out with the remaining 24 items. Tabachnick and Fidell [85] stated that a sample size of 200 would not violate normality in multivariate analyses. Accordingly, no normality examination was performed, and only Mahalanobis distance analysis was performed to control extreme values. After the analysis, 46 extreme values were removed from the data set and the analyses were continued with 224 data. Since the data set to be analyzed was a Likert-type scale [90], CFA was performed using the maximum likelihood estimation procedure. CFA was performed both at the first-level multidimensional and second-level multidimensional levels. In the examination of the fit values related to the structure in CFA, the following fit index values were taken as criteria: Chi-square (χ2) and Chi-square/degree of freedom (χ2/df < 3), comparative fit index (CFI > 0.95–0.90), root mean square error of approximation (RMSEA<0.05–0.08) [91] and standardized root mean square error of approximation (SRMR<0.05–0.10) (Brown and Cudeck, 1993; as cited in: [92] recommended for a sample size less than 250. The analyses were made using the AMOS 21 program.

## Results

### Findings of exploratory factor analysis (Study 1)

After the analyses conducted within the scope of Study 1, the KMO and Bartlett values of the remaining 25 items were re-examined in terms of their suitability for factor analysis, and it was determined that the number and distribution of the obtained data were suitable for EFA (KMO = .849, χ^2^ = 7951.437; sd = 300; p < 0.01) [82]. Five sub-dimensions with eigenvalues greater than 1 were identified in the 25-item scale. In EFA, factors with an eigenvalue of 1 or greater are considered as important [83]. According to the Scree Plot graph in Fig 3, five breakpoints with an eigenvalue of 1 or greater indicate that the scale forms a five-factor structure. The sub-dimensions, respectively, are named as “Visibility” (8 items), “Escape” (6 items), “Socialization” (4 items), “Reference” (3 items) and “Achievement” (4 items) (Table 1). The total variance explained by the five factors is 62.696 percent. 27.414% of the total variance is explained by the 1^st^ factor, 12.820% by the 2^nd^ factor, 10.086% by the 3^rd^ factor, 6.545% by the 4^th^ factor and 5.831% by the 5^th^ factor (Table 1). The total variance explained by the scale is above 50% [84] indicates that it is at the desired level of adequacy. Factor loadings of the items were determined to be between 0.96 and 0.60, and above 0.40 [85]. Cronbach’s Alpha internal consistency coefficient was used to determine the reliability of the scale in EFA analyses. The Cronbach Alpha internal consistency coefficient calculated for 25 items in the obtained data was.847. This value indicates that the scale has an acceptable internal consistency. The Cronbach’s Alpha coefficient for the sub-dimensions of the scale is.944 for the 1^st^ factor (Visibility),.861 for the 2^nd^ factor (Escape),.848 for the 3^rd^ factor (Socialization),.851 for the 4^th^ factor (Reference), and.784 for the 5^th^ factor (Achievement) (Table 1). Internal consistency coefficients at the sub-dimensions are also within acceptable ranges [93]. Table 2 shows that the item means range from 1.221 to 3.476 and the mean of all items is 2.039. Büyüköztürk [81] states that items with an item-total correlation of 0.30 and above are highly discriminatory, and items between 0.20 and 0.30 can be included in the test if deemed necessary. The item-total correlation of all items on the scale, except M25, is above 0.30, and M25 is within the acceptable range stated by Büyüköztürk [81].

**Table 1:** Results of Exploratory Factor Analysis of the YMS.

Factor	Item	Factor Loadings	Eigenvalue	Explained Variance	α
Visibility	M31	0.96	6,854	27,414	0.94
	M35	0.93			
	M32	0.92			
	M34	0.92			
	M30	0.87			
	M29	0.83			
	M28	0.62			
	M26	0.60			
Escape	M11	0.82	3,205	12,820	0.86
	M8	0.81			
	M7	0.76			
	M6	0.67			
	M10	0.64			
	M9	0.61			
Socialization	M43	0.84	2,521	10,086	0.84
	M42	0.79			
	M44	0.74			
	M45	0.69			
References	M37	0.91	1,636	6,545	0.85
	M36	0.85			
	M38	0.65			
Achievement	M23	0.78	1,458	5,831	0.78
	M25	0.68			
	M22	0.67			
	M24	0.60			

**Table 2:** Mean, Standard Deviation, Cronbach’s Alpha (α) and Item-Total Correlation Values.

Item	x̄	sd	item-total correlation	Cronbach’s alpha if item deleted
M31	1,22	0.41	0.45	0.84
M35	1,22	0.41	0.45	0.84
M32	1,23	0.42	0.46	0.84
M34	1,23	0.42	0.43	0.84
M30	1,27	0.45	0.44	0.84
M29	1,26	0.44	0.49	0.84
M28	1,39	0.55	0.47	0.84
M26	1,42	0.56	0.46	0.84
M11	2,39	1.14	0.47	0.83
M6	1,87	0.78	0.43	0.84
M7	1,96	0.86	0.47	0.83
M8	2,43	1.13	0.45	0.83
M9	2,70	1.11	0.39	0.84
M10	2,63	1.20	0.43	0.84
M42	1,73	0.82	0.43	0.84
M43	1,73	0.80	0.46	0.83
M44	2,73	1.20	0.37	0.84
M45	2,41	1.13	0.45	0.83
M36	2,41	1.13	0.30	0.84
M37	2,37	1.12	0.33	0.84
M38	2,14	1.00	0.39	0.84
M22	2,05	0.97	0.40	0.84
M23	2,23	1.04	0.45	0.83
M24	3,38	1.02	0.32	0.84
M25	3,47	1.02	0.27*	0.84
Overall Average	2.04			

**Fig 3:**
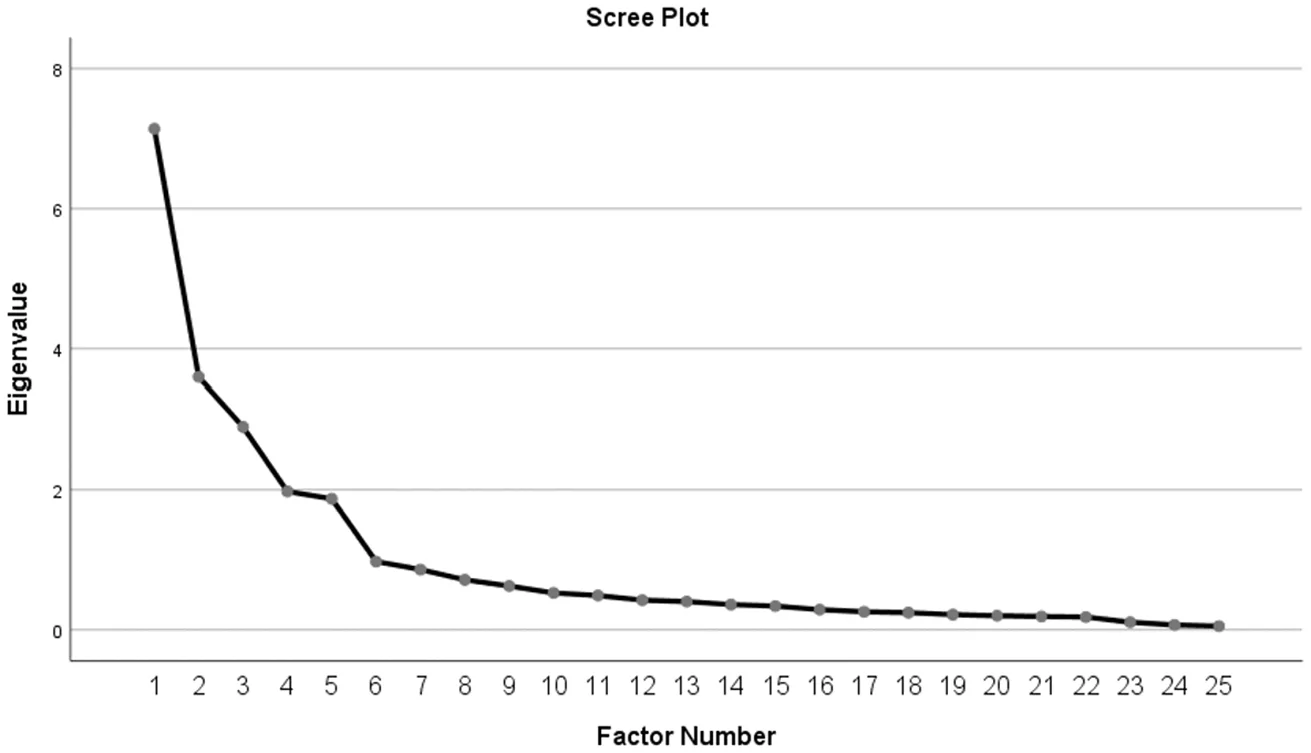
Scree plot graph obtained as a result of EFA.

### Findings of confirmatory factor analysis (Study 2)

In Study 2, confirmatory factor analysis of the 5-dimensional scale resulting from EFA was conducted twice: first-level multifactorial and second-level multifactorial. Table 3, Figs 4 and 5 show the modifications for the first and second level multifactorial confirmatory factor analysis and the goodness of fit values resulting from the modification.

**Table 3:** First and Second Level Multifactor Confirmatory Factor Analysis Goodness of Fit Values Before and After Modification.

	Critical values of goodness-fit indices	1	2	1	2
χ2		623.905	578.108	645.379	565.446
df		242	241	246	244
p		0.000	0.000	0.000	0.000
χ2/df*	<3	2.578	2.399	2.623	2.317
RMSEA*	<0.05-0.08	0.084	0.079	0.085	0.077
CFI*	>0.95-0.90	0.916	0.926	0.913	0.930
SRMR**	<0.05-0.10	1.027	0.078	1.054	0.078

**Fig 4:**
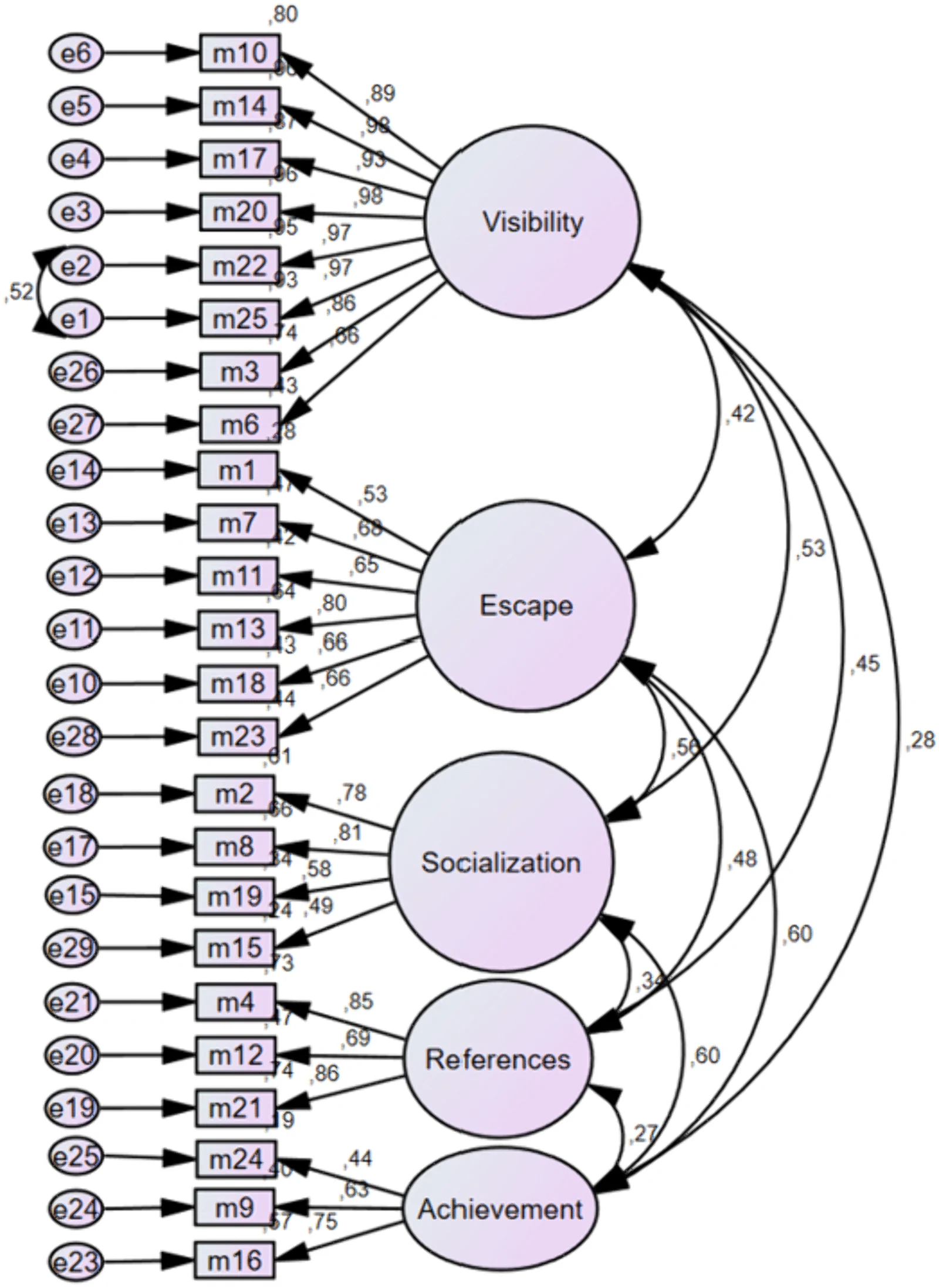
Single-Level Multifactor Model.

**Fig 5:**
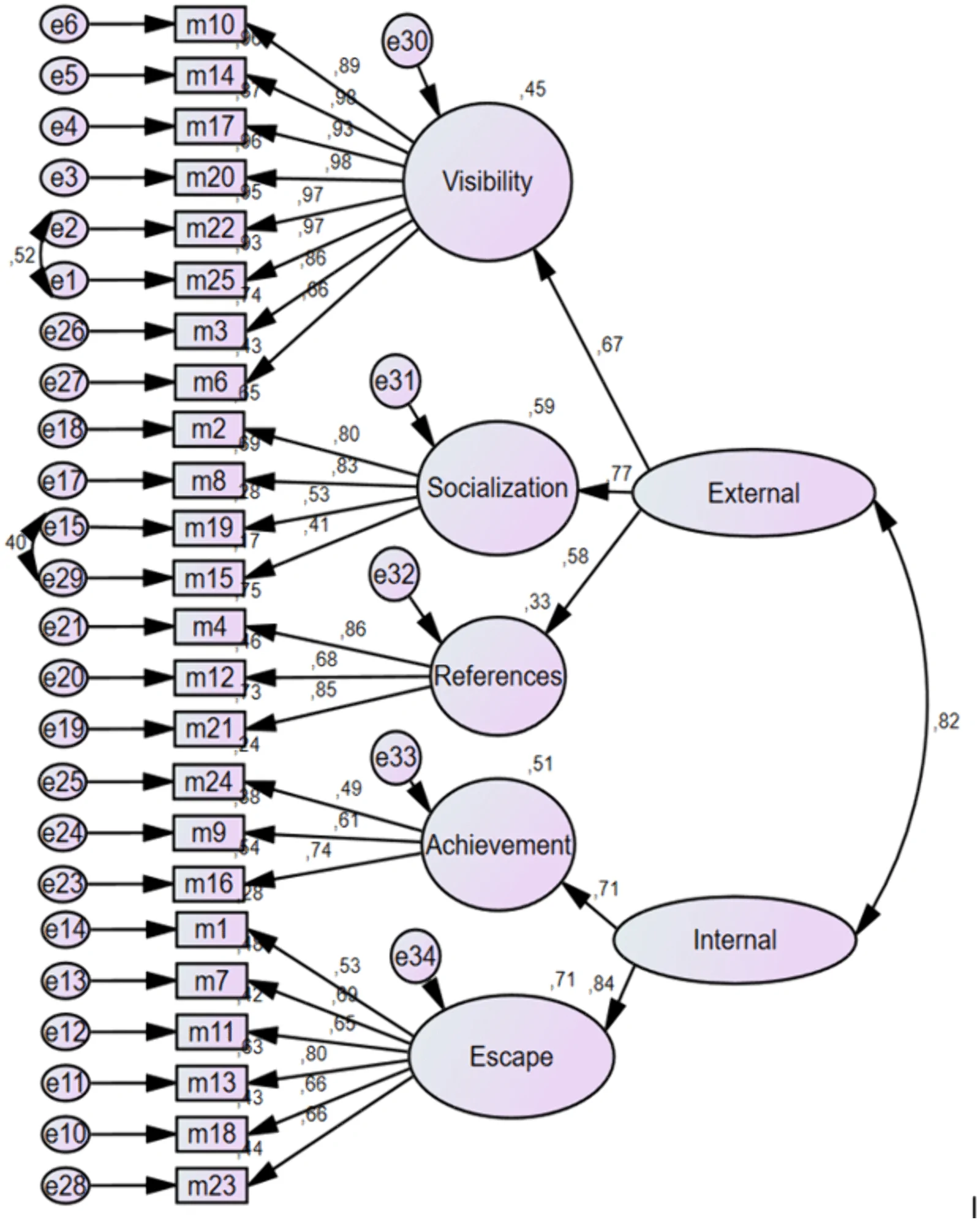
Double-Level Multifactor Model.

As stated in Table 3, since the RMSEA and SRMR values in the first-level multi-factor analysis were above the criterion index value range (RMSEA > 0.80 and SRMR > 0.80), modification suggestions were considered. Following the relevant suggestion, covariance was plotted between items 22 and 25, and the analysis was repeated (Fig 4). After the analysis, it was determined that the relevant values were within the acceptable range of the criteria index values. The same process was carried out for the bi-level scale as well. At this stage, after the first confirmatory factor analysis, the RMSEA and SRMR values were again not within the criterion index value range (RMSEA > 0.80 and SRMR > 0.80), so modification suggestions were examined. The covariance paths between Items 22 and 25 and between Items 15 and 19 were incorporated into the measurement model and are presented in Fig 5. Items 22 and 25 belong to the **Visibility** dimension, whereas Items 15 and 19 belong to the **Socialization** dimension. Within each dimension, these item pairs reflect conceptually similar aspects of the same latent construct. Therefore, the error terms of these items were allowed to covary, as they represent theoretically related content within their respective dimensions.After the second analysis, it was determined that all values were at an acceptable level within the criterion index value range (Table 3). As a result of these analyses, it was determined that the relevant scale can be used both as a five-dimensional scale and as a two-dimensional scale at the second level. In the first-level multifactorial model, it was determined that the factor loadings of the items varied between.98 and.43 (>0.40), t values varied between 59.661 and 5.161 (>1.96), R^2^ (Square of multiple correlation) values varied between 0.19 and 0.96, and corrected item-total correlation values varied between 0.37 and 0.95 (>0.30) (Table 4). According to the first-level multifactorial analysis, the Cronbach Alpha internal consistency coefficient for the whole scale (24 items) was calculated as 0.90 and Omega as 0.89. These values state that the scale has an accepted internal consistency [84,93,94]. As Table 5 is examined, it is seen that there is a positive moderate relationship between the factors in the first-level multifactorial model (p < 0.01). The analysis results for the verification of the second-level multifactorial model state that factor loadings ranged between.98 to.41 (>0.40), t-values ranged between 59.657 to 5.412 (>1.96), R^2^ (Square of Multiple Correlation) values ranged between 0.171 to 0.96, and corrected item-total correlation values ranged between 0.37 to 0.95 (>0.30) (Table 6). According to the second-level multifactorial analysis, the Cronbach Alpha internal consistency coefficient for the whole scale (24 items) was calculated as 0.90 and Omega as 0.89. These values state that the scale has an accepted internal consistency [84,93,94]. Cronbach’s Alpha coefficient for the sub-dimensions of the scale varies between.63 and.96, and Omega values vary between 0.64 and 0.96. It is seen that all the values specified in Table 6, comply with the criterion values within the literature [77,82,95]. Table 7 shows the correlation between the external and internal dimensions in the second-level multifactorial model and the other sub-dimensions under these dimensions. According to Table 7, there is a positive significant relationship between visibility, socialization and references, which constitute the external dimension, and escape and achievement which constitute the internal dimension, as well as between the internal and external dimensions (p < 0.01). The results of the final form of YMS are presented in Tables 8.

**Table 4:** Analysis Results for Validation of First -Level Multi-Factor Model.

Factor	M		Factor	t Value	R2	Corrected Item Total Correlation	α	Ω
**Visibility**	3	I do yoga because I really like its clothes	0.86	22.82	0.74	0.85	0.96	0.96
	6	I do yoga so that those around me can see that I am a pioneer in yoga	0.65	12.60	0.43	0.66		
	10	I do yoga to share on social media	0.89	26.07	0.79	0.89		
	14	I do yoga to show off to others	0.97	43.70	0.95	0.95		
	17	I do yoga because it’s in fashion	0.93	31.30	0.86	0.91		
	20	I do yoga to get more likes on social media	0.98	44.19	0.96	0.95		
	22	I do yoga to attract other ‘s attention	0.97	59.66	0.94	0.95		
	25	I do yoga to get more followers on social media	0.96	59.66	0.93	0.94		
**Escape**	1	I do yoga to escape from the city crowds.	0.52	7.05	0.27	0.49	0.82	
	7	I do yoga to escape my financial problems	0.68	7.05	0.46	0.56		0.82
	11	I do yoga to relieve my unhappiness	0.65	6.86	0.42	0.57		
	13	I do yoga to escape from problems at work	0.80	7.60	0.64	0.71		
	18	I do yoga to escape from environmental problems	0.65	6.89	0.43	0.63		
	23	I do yoga to escape from political problems	0.66	6.92	0.43	0.57		
**Socialization**	2	I do yoga to meet with new people	0.78	10.99	0.61	0.59	0.75	0.73
	8	I do yoga to have a social environment	0.81	10.99	0.65	0.57		
	15	I do yoga to be with people who feel in the same way	0.48	6.78	0.23	0.75		
	19	I do yoga to be together with people who do yoga	0.58	8.16	0.34	0.67		
**References**	4	I do yoga because my doctor referred	0.85	10.75	0.73	0.75	0.83	0.85
	12	I do yoga because my psychotherapist referred	0.68	10.75	0.47	0.61		
	21	I do yoga because my physiotherapist referred	0.85	13.18	0.73	0.74		
**Achievement**	9	I do yoga because it provides self-confidence	0.63	5.16	0.40	0.46	0.63	0.65
	16	I do yoga to satisfy my sense of of achieving something	0.75	5.30	0.56	0.51		
	24	I do yoga because of my ambition to achieve yoga poses	0.43	5.16	0.19	0.37		

α: 0.90 (24 items).

Ω: 0.89 (24 items).

**Table 5:** Correlation Analysis Between Sub-Dimensions of the First-Level Multifactor Model.

	Visibility	Escape	Socialization	References	Achievement
Visibility	1				
Escape	0,417**	1			
Socialization	0,528**	0,558**	1		
References	0,454**	0,477**	0,341**	1	
Achievement	0,284**	0,596**	0,597**	0,271**	1
**p < .01					

**Table 6:** Analysis Results for Validation of Second-Level Multifactor Model.

2^nd^ Level Factor	1^st^ Level Factor	M	Factor loading	t Value	R2	Corrected Item Total Correlation	α	Ω
**EXTERNAL** α=0.89; Ω=0.86	Visibility	3	0.86	22.83	0. 74	0.85	0.96	0.96
		6	0.65	12.61	0.43	0.66		
		10	0.89	26.09	0.79	0.89		
		14	0.97	43.65	0.95	0.95		
		17	0.93	31.31	0.86	0.91		
		20	0.98	44.15	0.96	0.95		
		22	0.97	59.65	0.94	0.95		
		25	0.96	59.65	0.93	0.94		
	References	4	0.86	10.63	0.74	0.75	0.83	0.85
		12	0.68	10.63	0.46	0.61		
		21	0.85	13.03	0.73	0.74		
	Socialization	2	0.80	10.80	0.64	0.59	0.75	0.73
		8	0.83	10.80	0.68	0.57		
		15	0.41	5.72	0.17	0.75		
		19	0.53	7.45	0.28	0.67		
**INTERNAL** α=0.82; Ω=0.81	Achievement	9	0.61	5.41	0.37	0.46	0.63	0.65
		16	0.73	5.59	0.54	0.51		
		24	0.48	5.41	0.23	0.37		
	Escape	1	0.52	7.07	0.27	0.49	0.82	0.82
		7	0.68	7.07	0.47	0.56		
		11	0.64	6.83	0.42	0.57		
		13	0.79	7.58	0.63	0.71		
		18	0.65	6.88	0.43	0.63		
		23	0.66	6.91	0.43	0.57		

α: 0.90 (24 item).

Ω: 0.89 (24 items).

**Table 7:** Correlation Analysis between Sub-Dimensions of the Second-Level Multi-Factor Model.

	Extarnal	Visibility	Socialization	References
Extarnal	1			
Visibility	0,869**	1		
Socialization	0,735**	0,441**	1	
References	0,690**	0,447**	0,272**	1
**p < .01				
	Internal	Escape	Achievement	
Internal	1			
Escape	0,935**	1		
Achievement	0,738**	0,451**	1	
**p < .01				
	External	Internal		
External	1			
Internal	0,824**	1		
**p < .01				

**Table 8:** Final Version of *YMS:* Renumbered items.

Numbers	Items
1	I do yoga to escape from the city crowds.
2	I do yoga to meet with new people.
3	I do yoga because I really like its clothes.
4	I do yoga because my doctor referred.
5	I do yoga so that those around me can see that I am a pioneer in yoga.
6	I do yoga to escape my financial problems.
7	I do yoga to build a social circle.
8	I do yoga because it gives me self-confidence.
9	I do yoga to share on social media.
10	I do yoga to get rid of my unhappiness.
11	I do yoga because my psychotherapist recommended it.
12	I do yoga to escape problems at work.
13	I do yoga to show off to others.
14	I do yoga to be with people who feel in the same way.
15	I do yoga to satisfy my desire to achieve something.
16	I do yoga because it’s trendy.
17	I do yoga to escape environmental issues.
18	I do yoga to be around people who do yoga.
19	I do yoga to get more likes on social media.
20	I do yoga because my physical therapist recommended it.
21	I do yoga to attract other people’s attention.
22	I do yoga to escape political issues.
23	I practice yoga because of my ambition to achieve yoga poses.
24	I do yoga to get more followers on social media.

Following all analyses, the final renumbered version of the 24-item scale is presented in Table 8. In addition, the allocation of the renumbered items to their respective subdimensions is provided in detail in Table 9.

**Table 9:** Final Version of *YMS:* Factors and items.

Factor	Factor	Items	Min. Score	Max. Score
**External**	Visibility	3,5,9,13,16,19,21,24	8	40
	References	4,11,20	3	15
	Socialization	2,7,14,18	4	20
**Internal**	Achievement	8,15,23	3	15
	Escape	1,6,10,12,17,22	6	30

## Discussion

Turkey is a new market for yoga. Information about the reasons and motivations of consumers in this market for purchase of yoga-related services/products is quite limited. It has been observed that yoga motivations are measured based on exercise, physical activity, and sports motivations [37,39,57] in existing studies in the literature. However, it is thought that there may be differences between the yoga motivation dynamics of the markets where these studies were conducted and the yoga motivations in the Turkish market, where yoga has just started to spread. The limited information on whether yoga is an activity based on sports, exercise, or physical activity in Turkey led the research team to develop a new yoga motivation scale with an exploratory approach.

In the first stage indicated in the flow diagram, the research group’s previous qualitative study and the existing literature served as a guide, and in order to avoid any gaps in the item pool, a draft scale was created that would include the motivation elements revealed in qualitative and quantitative studies conducted both in other countries and in Turkey. After the content validity study, 51 items remained on the scale. After this stage, various analyses were utilized for purification and verification of the scale in two consecutive independent studies. EFA analysis in Study 1 revealed the structure of the YMS consisting of 25 items and 5 dimensions. In Study 2, which was conducted to test the validity and reliability of the structure that emerged after EFA, CFA and Cronbach’s Alpha and Omega internal consistency coefficient reliability analysis were applied. CFA was applied at both first-level and second-level. In the correlation analysis related to the sub-dimensions of the YMS consisting of 24 items and 5 dimensions after CFA, a moderately significant relationship was detected. Second-level CFA has shown that 6-item “Escape” and 3-item “Achievement” dimensions are internal, whereas 8-item “Visibility”, 4-item “Socialization” and 3-item “References” dimensions are external motivation elements. As can be seen in the findings section, a valid and reliable measurement tool was obtained as a result of the study.

The visibility dimension of yoga participation motivations is a dimension that is not included in quantitative studies conducted to determine yoga motivations and is shaped as an external motivation source in the current study. The items in the visibility dimension are related to being visible on social media and the fashion aspect of yoga. According to Schnäbele [96], as cited in Smith and Atencio [97], yoga is defined “by the visibility of the consuming body within consumer society, consuming food, fashion, attitudes, and bodily techniques”. Jain [11] states that Instagram, one of the social media tools, has become a modern tool for yoga and yoga entrepreneurs to “package” the practice. Although this is not the case in other quantitative studies, some qualitative studies, in particular, touch on the relationship between yoga and social media. While Mora et al. [16] state that the global-scale growth of the yoga industry is largely driven by social media, Malenović [98] emphasized that millions of people share yoga visuals on social media for every situation, body type, age, and location. Cowans [99] specifically points out that Instagram has become a place where yogis of all kinds can virtually share their favorite poses, sequences, and yoga philosophies.Yoga consumers (“modern yogis”) share their experiences and gain prestige by participating in yoga events through Instagram posts [72]. Many yoga consumers enjoy sharing photos of their yoga poses with their friends on WeChat after taking a yoga class [21]. Lacasse, Santarossa, & Woodruff [100] stated that the highest motivation was promotion in their study on Instagram to understand whether the posts of yoga students and teachers were consistent with the fundamental reasons for practicing yoga.It is stated that yoga participants in Turkey use multiple platforms to express their ideas about yoga, being a yogi or yogini, and how a yogi or yogini should live, and they have a common concern about social visibility, and that this concern is strengthened by the way they use social media [101]. Social media has become a place where yoga instructors and students reflect their own presentations, promote products and services related to the yoga sector, and share their collaborations with brands [102]. In addition to reasons such as the health industry and accessibility of yoga, it is stated that the fact that it is fashionable, people who do yoga tell their friends about their passion for yoga, share yoga information on social media, write blogs and large companies selling yoga products contribute to this situation [51]. Castle [103], the social media manager of Yoga for Health & Education Trust, states that based on the advocacy of yoga for everyone, more content will be seen on social media about various topics and products related to yoga. Based on all these, it becomes understandable that visibility emerges as a dimension among the motivation sources of yoga participants.

Another source of external motivation is socialization. This dimension includes items such as making a social circle, meeting new people, and being together with yoga practitioners. In this scale that we developed specifically for yoga, socialization is seen to be a motivation, similar to other scales [37,57]. However, the scales used in other studies are the Sport Motivation Scale, and the Physical Activity and Leisure Motivation Scale. Socialization is one of the basic motivations of sports consumers [104], and it was defined as a yoga motivation due to its presence in the scales used in the other two studies. However, in our study, it was understood that socialization is a direct reason for participation specific to yoga. The items in the references dimension show that the participants in this study did yoga due to the advice of different health experts, and this was an external source of motivation. In studies where they collected data through surveys, Büssing [105] stated that approximately 49% of yoga participants started practicing after receiving advice from a health professional, while Park et al. [20] stated that advice from a health professional, among “other sources of motivation”, was a guide in participating in yoga. Brems et al. [44] found that 19% of yoga participants were motivated by guidance and that guidance from healthcare providers was the lowest-scoring motivation source. It is thought that since yoga has a shorter history in Turkey compared to many countries in the world [53] and is not yet a practice accepted by the general public [52], people may need references more, and this constitutes one of the dimensions of the scale. It is also consistent with the information that consumers may be open to the influence of reference groups, especially in cases where they do not have much knowledge about a product [106], and that the influence of reference groups is significantly persuasive in the adoption of new products [107]:15).

It was found that the YMS has two dimensions related to escape and achievement and that these were located under the internal motivation dimension in the two-level CFA analysis. The escape dimension is related to yoga participants’ escape from problems such as work, environment and economy. These types of problems are among the main stress sources of today’s city dwellers and people are looking for ways to get away from their problems. We can say that one of the reasons why one of the motivations for participation in yoga is escape is this, based on studies reporting stress management as a yoga motivation [20,36,39,43,46,49,51,108]. However, we would like to draw attention to the following; in this study, it was observed that the items initially determined regarding spirituality did not work and that spirituality was not shaped as a dimension of the scale. However, it may be possible to associate the “escape” dimension, which is among the internal motivations, with spirituality. According to Iso-Ahola’s motivation paradigm, spiritual tourists escape from noise, people, routine, materialism, material comfort and inner emptiness and seek loneliness, loss of self, self-focus, self-reliance, silence, etc., and may seek solitude and peace by escaping from people and noise in order to find inner peace or answers to their existential questions [109].

The achievement dimension includes items related to the satisfaction of the sense of achievement and the ambition to do yoga poses. In the study where [39] adapted the Exercise Motivation Inventory-2 (EMI-2), the challenge is a dimension similar to achievement in terms of structure. Despite being a new market, the fact that yoga participants from Turkey participated in the World Yoga Championship and won 16 gold, 14 silver, and 14 bronze medals, making a total of 44 medals [110] can be considered as evidence that yoga consumers in Turkey have a success-oriented orientation.

## Conclusions, limitations and suggestions

The current Yoga Motivation Scale (YMS) was developed in a market where yoga recognition, awareness, and participation are relatively low. The limited information about this market led to the development of a new scale instead of the scales used in other studies with the aim of understanding the structure of the market. As a result, this study develops and validates a five-factor YMS in an emerging market, identifying a novel visibility dimension reflecting social-media/status-related motives alongside escape, achievement, socialization, and reference. Also, this scale, which we developed specifically for the Turkish market, revealed that there are internal and external motivation elements in yoga participation. Our scale has shown that although it has similar motivations to those of the consumers in markets where yoga has been practiced for a longer time, it does not include basic motivations such as spirituality, health, or exercise, which are included in other scales. This can be considered a limitation of our study. Spirituality is defined as a continuation motivation in some studies [39]. Approximately 57% of those in our study in EFA (less than 1 year, 17% + 40.6% between 1–3 years), and approximately 65.6% in CFA (less than 1 year, 34.8% + 30.8% between 1–3 years) declared that they have been practicing yoga for a short time. Despite the large sample size, the number of participants who have been practicing yoga for a short time is striking, and it is seen that the motivations in the scale are shaped around this situational feature. It is seen that spirituality does not become apparent in an environment where continuity is not provided, and in fact, the results presented in this direction in the literature are also supported. Furthermore, findings from studies related to social media, indicating that the spiritual aspect of yoga does not emerge [100] or remains limited to rates such as 5% [111], can be considered as factors preventing the emergence of spirituality in this study. Apart from this, it is thought that the unstructured dimensions such as health and exercise may be related to the low level of inactivity in adults in Turkey [112,113].

The fact that the study data were collected during the COVID-19 period can be considered a limitation. The COVID-19 pandemic marked a time when people were confined to their homes and spending a significant portion of their time on social media [114,115]. It is known that people communicated through social media [116] and used it extensively during this period. Even before COVID-19, a stream of over 15 million posts emerged on social media (specifically Instagram) with the #yoga hashtag, a number reported to be increasing daily [99]. The increase in yoga-related posts even before the COVID-19 era, and the likelihood of this continuing during the pandemic, may be related to the emergence of the “visibility” dimension on the developed scale.

Considering the subjectivity of consumers’ purchasing motivations [61] and the duration of yoga experience [46], it can be thought that the results of this scale developed in the Turkish market cannot be generalized.One of the limitations of the present study is that the sample predominantly consisted of Turkish women and individuals who were novice yoga practitioners. This may limit the generalizability of the findings to other demographic groups and practitioners with different levels of yoga experience. Accordingly, this should also be considered an important limitation in the Turkish context. Future studies are therefore encouraged to include more balanced and heterogeneous samples in terms of gender and yoga experience, which may enhance the generalizability and external validity of the findings. However, it is important to underline that yoga can work in similar markets where it has just started/will start developing. It is thought that the current scale will be an important tool in assessing the purchasing motivations of yoga consumers in order to reach new markets and popularize yoga, which is said to be an export product [30]. However, as [39] point out, it is possible that different motivations may exist for different yoga consumer groups (exercise-oriented, yogis and postural yogis). Therefore, it is recommended that this limitation be recognized in future studies and, if necessary, a separate scale be developed and implemented for each group. At the same time, it may be advisable to conduct analyses such as test-retest reliability and multi-group invariance in future studies.

## Supporting information

S1 File Information on data uploaded to the Dryad repository and assigned a DOI. (ZIP)
