# Supplementary material for: Turkish yoga consumers’ motivation: A scale development study
Source: PLoS One. 2026 Oct 9;21(10):e0359209. doi: 10.1371/journal.pone.0359209 (PMC13652277; doi:10.1371/journal.pone.0359209)
Supplement: Supplementary file 1 [file pone.0359209.s002.docx]

We sincerely appreciate the reviewers’ insightful suggestions and constructive feedback, which have greatly contributed to the improvement of our manuscript. In the following sections, we provide detailed responses to each of the reviewers’ comments, together with the corresponding revisions incorporated into the manuscript.

Reviewer-1

| Comments and Suggestions | Responses |
| --- | --- |
| 1- Origins of yoga: The sentence “Yoga originated among the lower castes…” is historically contested and unnecessarily provocative. Replace with a neutral, mainstream formulation (e.g., roots in ancient South Asian traditions; classical sources like Patañjali; transmission to the West via 19th–20th century interlocutors). | The relevant sentence has been changed as follows:  It is derived from the root yoga Yuj, which has a long history of 5000 years before it emerged in India. This ancient Indian practice was first devised by Rishis (sages) in search of happiness and became known as yoga, embodying the concept of unity between body, mind and soul. ( line 45-47) |
| 2- “Purchasing yoga” phrasing: Use “purchase of yoga-related services/products” or “participation/consumption of yoga services” to avoid conceptual confusion between practice and commodity. | The relevant phrasing has been changed as follows:  Purchase of yoga-related services/products (line 33, line 404 ) |
| 3- Contribution: Make explicit what is new versus prior motivation scales (e.g., EMI-2 adaptations). Highlight that “Visibility” (social-media/fashion/status) emerges distinctly in a new/early-stage market—this is the differentiator. | Line 425 – 427; 428-430; 433-437; 440-442 of the article, we used the following statement:  *“The visibility dimension of yoga participation motivations is a dimension that is not included in quantitative studies conducted to determine yoga motivations and is shaped as an external motivation source in the current study”*  According to Schnäbele (2013), as cited in Smith and Atencio (2017), yoga is defined "by the visibility of the consuming body within consumer society, consuming food, fashion, attitudes, and bodily techniques”.  While Mora et al. (2018) state that the global-scale growth of the yoga industry is largely driven by social media, Malenović (2016) emphasized that millions of people share yoga visuals on social media for every situation, body type, age, and location. Cowans (2016) specifically points out that Instagram has become a place where yogis of all kinds can virtually share their favorite poses, sequences, and yoga philosophies.  Lacasse, Santarossa, & Woodruff (2019) stated that the highest motivation was promotion in their study on Instagram to understand whether the posts of yoga students and teachers were consistent with the fundamental reasons for practicing yoga |
| 4- Gender skew (EFA 94.9% female; CFA 89.3% female) + self-selection + COVID-era online collection → acknowledge bias and limit generalization. Add rationale for recruiting channel and how it may inflate “visibility/socialization” items. Provide response flow (invited/started/valid) and a brief nonresponse bias check if possible. | The high prevalence of the female population in both the EFA and CFA regarding the gender skew is consistent with the information presented in the rationale of our study. Therefore, since the gender skew is similar to that of other studies, it was not specified as a limitation (Line 116-119)  The following explanations regarding COVID-19 have been added to the conclusion section. (Line 524-531)  “The fact that the study data were collected during the COVID-19 period can be considered a limitation. The COVID-19 pandemic marked a time when people were confined to their homes and spending a significant portion of their time on social media (Kaya, 2020; Yıldırım, 2020). It is known that people communicated through social media (Tsao et al., 2021) and used it extensively during this period. Even before COVID-19, a stream of over 15 million posts emerged on social media (specifically Instagram) with the #yoga hashtag, a number reported to be increasing daily (Cowans, 2016). The increase in yoga-related posts even before the COVID-19 era, and the likelihood of this continuing during the pandemic, may be related to the emergence of the "visibility" dimension on the developed scale.” |
| 5-Psychometrics: report fully and tighten analyses | Table 1 added. |
| 6- EFA: Present a table with item-level loadings, cross-loadings, communalities, uniqueness, eigenvalues, and variance explained. Your text says loadings .96–.60; show the full matrix and the exact retention rules applied at each pruning step. Factor retention: Besides eigenvalues>1, add parallel analysis and scree justification.  Rotation: Oblimin is fine; state δ if non-default. | It is shown in Table 2. |
| 7- CFA: Report full fit indices with exact values: χ²(df), χ²/df, CFI, TLI, RMSEA (90% CI), SRMR. You currently state they “met criteria” but do not present the numbers. | Table 3 added. |
| 8- Estimation: With 5-point Likert, robust ML (MLR) or WLSMV is preferable to plain ML. Report normality checks; if non-normal, rerun with robust estimator or justify ML with simulation references. | Line 216-218 and 323-328 of the article, we used the following statement:  *Before the EFA process, it was examined whether the data was normally distributed, and it was determined that the Skewness and Kurtosis values (Skewness -1.106-1.352; Kurtosis -1.741-1.477) were between -2 and +2 (George and Mallery, 2010), which are accepted in the literature.*  *Tabachnick and Fidell (2001) stated that a sample size of 200 would not violate normality in multivariate analyses. Accordingly, no normality examination was performed, and only Mahalanobis distance analysis was performed to control extreme values. After the analysis, 46 extreme values were removed from the data set and the analyses were continued with 224 data. Since the data set to be analyzed was a Likert-type scale (Gürbüz, 2019), CFA was performed using the maximum likelihood estimation procedure.* |
| 9- Second-order model: You interpret Intrinsic (Escape, Achievement) and Extrinsic (Visibility, Socialization, Reference) via a two-level CFA. Report 2nd-order loadings, compare fit to the correlated 5-factor model (Δχ², ΔCFI/SRMR), and justify the higher-order structure theoretically. | Table 7 was added. |
| 10. Reliability: Report omega (ω) in addition to alpha, and composite reliability (CR) + AVE per factor. Demonstrate convergent (AVE ≥ .50) and discriminant validity (Fornell-Larcker, or HTMT with thresholds). | Omega values were reported (Table 4, Table 6) |
| 11- Common method bias: Add a brief check (e.g., Harman’s single-factor, or a latent method factor sensitivity). | As we could not fully clarify the scope of the analysis requested by the reviewer, we were unable to implement it. |
| 12- Item content validity: You used Lawshe CVR with 9 experts (cut-off .75). Report per-item CVR, S-CVI/Ave, and inter-rater agreement (if computed). | Inter-rater reliability was calculated according to Lawshe. Line 225-228 of the article, we used the following statement:  *According to Lawshe's (1975) content validity index, the minimum value required for 9 experts in terms of consensus is 0.75. 67 items of the 118-item draft scale were eliminated because they were below 0.75, and 51 items with a value of 0.77-1 on which the experts reached a consensus remained.* |
| 13. Include Appendix with: (a) final 24 items grouped by factor; (b) instructions & response scale; (c) scoring (sum/mean per subscale and optional overall intrinsic/extrinsic), (d) interpretation guidance (no norms yet → report sample means/sds and suggested percentile bands). | a. Table 8 has been added.  b. Line 216-218 of the article, we used the following statement:  “The use of the "Five-Point Likert Type" measurement tool was preferred for the scale, and the statements were rated between 5 and 1 between "strongly agree" and "strongly disagree".”  c. Table 8 has been added.  d. Information regarding the sample is provided in the data collection and sample sections of the study. However, it is not presented in a table format. |
| 14. Clarify language: if items were in Turkish, document translation/back-translation and any cognitive interviewing/pilot for clarity. | Since this is not a scale adaptation study, there is no translation-back translation phase. Table 6 contains the English translations of the Turkish scale items. The translations were performed by a language expert. |
| 15. Indicate time to complete, missing-data handling, and whether there are reverse-scored items (currently none—consider adding 1–2 to reduce acquiescence bias in future versions). | Line 213- 216 of the article, we used the following statement:  In creating the initial item pool, the items to be included in the scale were discussed and prepared by the researchers, taking into account the suggestions made by DeVellis (2017:73-85), short sentences were used in the item writing, the number of items was increased by writing alternative items in order to find the item that would best express the concept, and negatively/reverse scored items were not written. |
| 16. You describe online consent; add an Ethics approval statement (Board name, approval no./date). Many journals require it even for anonymous surveys. Add data protection steps (storage, de-identification, access control).13. | Line 240-241 of the article, we used the following statement:  *Data were collected after obtaining ethical approval from the Human Research Ethics Committee (Protocol No. 2021/92).*  Note: At the request of the journal editor, the name of the university is not disclosed; the ethics approval document has been uploaded to the journal’s editorial system. |
| 17. Add concise descriptive stats (means/sds) for each factor in both samples; provide inter-factor correlations with 95% cıs.  If you drew modification indices between item errors (e.g., items 22↔25; 15↔19), show substantive justification (similar wording, content overlap) and confirm they are within-factor. Avoid cross-factor correlated errors. | Table 1 and Table 3 could be seen. |
| 18. Terminology consistency: Türkiye vs Turkey vs Turkiye—pick one (journals often prefer Türkiye in national context, Turkey in English manuscripts). | All relevant terms have been corrected/updated to Turkey. |
| 19. Dates & sources: Many citations are 2025 and web-based (organisations, blogs). Ensure authoritative sources and stable urls/doıs; avoid non-scholarly claims (e.g., exact “300 million participants” or “$338B industry”) unless triangulated with reputable industry reports or peer-reviewed sources; otherwise label as market estimates. International Yoga Federation descriptions and “Yoga as sport” claims: ensure accurate, citable details. | Some sources are dated 2025. For example, Deslippe (2019), Basavaraddi (2015).  In addition, since academic studies on yoga are quite limited, internet-based sources had to be used in the introduction section. |
| 20. Language edits: Replace value-laden phrasing (“no continuity of ancient traditions”) with neutral academic tone. | The phrase “no continuity of ancient traditions” that you mentioned does not appear in our text. |
| 21. Table 1: EFA loadings (+ communalities).  Table 2: CFA fit indices (baseline and post-modification). Table 3: CFA standardized loadings (λ), SE, z, R². Table 4: Factor correlations (EFA and CFA). Figure 3/4: Clean path diagrams (standardized). | The factor loadings were presented in Table 2.  Path diagrams were standardized (Figure 4 and 5)  First- and Second-Level Multifactor Confirmatory Factor Analysis Fit Indices Before and After Modification were added (Table 3).  Factor correlations (EFA and CFA) were added (Table 5 and Table 7) |
| 22. Limitations: Add (a) non-probability sampling, (b) gender skew, (c) online pandemic context, (d) lack of test–retest, (e) no criterion validity (e.g., correlations with related constructs such as exercise identity, social media use, or stress). | a) Line 253-254 and 307 -308 of the article, we used the following statement:  At this stage data was initially collected from 667 individuals using the convenience sampling method.  As a result of the EFA, data was collected from 270 people by using convenience sampling method to verify the validity of the scale consisting of 5 dimensions and 25 items.  b. The high prevalence of the female population in both the EFA and CFA regarding the gender skew is consistent with the information presented in the rationale of our study. Therefore, since the gender skew is similar to that of other studies, it was not specified as a limitation (Line 116-119)  C) The fact that the study data were collected during the COVID-19 period can be considered a limitation. The COVID-19 pandemic marked a time when people were confined to their homes and spending a significant portion of their time on social media (Kaya, 2020; Yıldırım, 2020). It is known that people communicated through social media (Tsao et al., 2021) and used it extensively during this period. Even before COVID-19, a stream of over 15 million posts emerged on social media (specifically Instagram) with the #yoga hashtag, a number reported to be increasing daily (Cowans, 2016). The increase in yoga-related posts even before the COVID-19 era, and the likelihood of this continuing during the pandemic, may be related to the emergence of the "visibility" dimension on the developed scale. (line 524-531)  d– At the same time, it may be advisable to conduct analyses such as test-retest reliability and multi-group invariance in future studies. (Line 542-543)  e) A suitable scale adapted to Turkish could not be found, so a criterion validity study could not be conducted. Also Line 184 and 187 of the article, we used the following statement: In the enquiries conducted, no scale developed for yoga motivations in Turkey was found. It was determined that the scale studies that could be reached were scales for behavioral elements other than motivation, such as the yoga self-efficacy scale adapted by Tetik Küçükelçi (2018) from Birdee et al. (2016); and the yoga belief scale adapted by Tetik Küçükelçi (2019) from Sohl et al. (2011). |
| 23. Future work: Recommend test–retest reliability, multi-group invariance (gender, experience <1y vs ≥1y, region), and predictive validity (does Visibility predict spend on apparel/memberships? Does Escape predict stress reduction outcomes?). | The relevant phrasing has been added as follows:  At the same time, it may be advisable to conduct analyses such as test-retest reliability and multi-group invariance in future studies. (Line 542-543)  The following sentence regarding visibility has been added.  Furthermore, findings from studies related to social media, indicating that the spiritual aspect of yoga does not emerge (Lacasse, Santarossa & Woodruff, 2019) or remains limited to rates such as 5% (Islam and Goldwasser, 2021), can be considered as factors preventing the emergence of spirituality in this study. (Line 518-521) |
| 24. Quick language fixes (illustrative) Title/abstract: Replace “purchasing yoga” with “purchasing yoga-related services/products or participating in yoga services.” | The relevant phrasing has been changed as follows:  Purchase of yoga-related services/products (line 33, line 404 ) |
| 25. Intro line: “Yoga, rooted in ancient South Asian contemplative traditions, integrates mental and physical practices oriented toward mind–body harmony.” | The relevant sentence has been changed as follows.  It is derived from the root yoga Yuj, which has a long history of 5000 years before it emerged in India. This ancient Indian practice was first devised by Rishis (sages) in search of happiness and became known as yoga, embodying the concept of unity between body, mind and soul. (line 45-47) |
| 26. Contribution sentence: “This study develops and validates a five-factor Yoga Motivation Scale (YMS) in an emerging market, identifying a novel Visibility dimension reflecting social-media/status-related motives alongside Escape, Achievement, Socialization, and Reference.” | This contribution sentence has been added in conclusion in line 504-506. |

Reviwer 2

1. In sports marketing, sports consumers are categorized into two groups: participants (active) and spectators (passive). Those who perform or practice any sports/exercise activity are defined as sports participants. Based on this framework, we have used the terms "consumer" and "participant" interchangeably for yoga practitioners where appropriate. We believe it is conceptually sound to evaluate yoga participants as a type of consumer, similar to general sports participants.
2. Intrinsic and extrinsic motivational factors are widely utilized in consumer behavior research. The following studies serve as significant examples of this approach:

Shang, R. A., Chen, Y. C., & Shen, L. (2005). Extrinsic versus intrinsic motivations for consumers to shop on-line. *Information & management*, *42*(3), 401-413.

Feng, X., Fu, S., & Qin, J. (2016). Determinants of consumers’ attitudes toward mobile advertising: The mediating roles of intrinsic and extrinsic motivations. *Computers in Human Behavior*, *63*, 334-341.

3. We would like to thank the reviewer for emphasizing the importance of measurement invariance. Due to sample size imbalances in certain subgroups (particularly regarding gender), conducting a full multi-group invariance test was statistically unfeasible. Consequently, we were unable to include the suggested analysis in our study.
4. We appreciate your recommendation. In line with your suggestion, we have added the following explanation to the "Limitations" section of our study:

“Although the present scale revealed motivational patterns comparable to those identified in markets where yoga has a longer tradition, it did not capture foundational motivations such as spirituality, health, or exercise that are included in other established instruments. The non-emergence of theoretically expected dimensions such as spirituality, health, and aesthetics constitutes an important limitation of the study. This finding should not be interpreted as evidence of the absence of these constructs; rather, it may reflect contextual influences (e.g., post-pandemic conditions), sample composition (predominantly beginner practitioners), or other measurement-related factors”.

5. The information you requested has been provided in **Table 3**.
6. In the "Item Reduction" section, details regarding normality were presented with references to established literature.
