# Supplementary material for: Turkish yoga consumers’ motivation: A scale development study
Source: PLoS One. 2026 Oct 9;21(10):e0359209. doi: 10.1371/journal.pone.0359209 (PMC13652277; doi:10.1371/journal.pone.0359209)
Supplement: Supplementary file 2 [file pone.0359209.s003.docx]

We sincerely appreciate the reviewers’ insightful suggestions and constructive feedback, which have greatly contributed to the improvement of our manuscript. In the following sections, we provide detailed responses to each of the reviewers’ comments, together with the corresponding revisions incorporated into the manuscript.

Reviewer-1

| Comments and Suggestions | Responses |
| --- | --- |
| 1- The sample composition raises concerns about the generalizability of the findings. There is a strong gender imbalance (EFA: 94.9% female; CFA: 89.3% female), along with a dominance of relatively inexperienced practitioners (over 57% with less than three years of experience in the EFA sample). This limits the applicability of the scale across broader yoga populations. | The following explanations have been added to the conclusion section:  One of the limitations of the present study is that the sample predominantly consisted of Turkish women and individuals who were novice yoga practitioners. This may limit the generalizability of the findings to other demographic groups and practitioners with different levels of yoga experience. Accordingly, this should also be considered an important limitation in the Turkish context. Future studies are therefore encouraged to include more balanced and heterogeneous samples in terms of gender and yoga experience, which may enhance the generalizability and external validity of the findings. |
| 2- Additionally, the use of convenience sampling through yoga centers may introduce self-selection bias, potentially overrepresenting individuals motivated by social or visibility-related factors. The manuscript does not include subgroup analyses (e.g., by gender or experience level), nor does it assess measurement invariance. Response rates and potential non-response bias are also not adequately addressed, particularly given the substantial amount of missing demographic data. | We thank the reviewer for this valuable comment. We acknowledge the limitation associated with the use of convenience sampling. However, data collection for the EFA phase was carried out during the COVID-19 pandemic, when government-imposed restrictions substantially constrained access to participants. Therefore, convenience sampling was adopted as the most practical data collection method. To enhance data quality, all responses were carefully screened, and incomplete or inconsistent cases were excluded from the analyses. |
| 3- While the factor analyses are generally well executed, several gaps remain. In the EFA, key diagnostics such as parallel analysis and scree plot interpretation are underreported. Extremely high factor loadings (up to 0.96) raise concerns about potential redundancy or suppression effects, and the manuscript does not provide full item-level statistics (e.g., communalities, uniqueness). | *Given the relatively high factor loadings (up to .96) observed for some items, we carefully reexamined the item pool for potential item redundancy and suppression effects. The results indicated that these items were theoretically and conceptually consistent with their respective latent constructs. Therefore, the high factor loadings were interpreted as reflecting a strong relationship between the items and the underlying constructs.* |
| 4- In the CFA, the use of maximum likelihood estimation assumes normality, but tests for this assumption are not sufficiently reported. Model modifications (e.g., correlated error terms) are introduced without clear theoretical justification. | The following explanations have been added to the findings section:  The covariance paths between Items 22 and 25 and between Items 15 and 19 were incorporated into the measurement model and are presented in Figure 5. Items 22 and 25 belong to the **Visibility** dimension, whereas Items 15 and 19 belong to the **Socialization** dimension. Within each dimension, these item pairs reflect conceptually similar aspects of the same latent construct. Therefore, the error terms of these items were allowed to covary, as they represent theoretically related content within their respective dimensions. (Line 361-367) |
| 5-The study also lacks additional validity checks, including convergent and discriminant validity (e.g., AVE, CR, HTMT), and does not address common method bias (e.g., Harman’s single-factor test). | We would like to thank the reviewer for emphasizing the importance of measurement invariance. Due to sample size imbalances in certain subgroups (particularly regarding gender), conducting a full multi-group invariance test was statistically unfeasible. Consequently, we were unable to include the suggested analysis in our study. |
| 6- Furthermore, the second-order factor model is mentioned but not adequately explained, particularly in terms of factor loadings and theoretical interpretation. The absence of test–retest reliability and criterion validity (e.g., comparison with existing scales) also limits the robustness of the validation. | Thank you for this valuable comment. The absence of test-retest reliability and criterion validity analyses had already been acknowledged in the Limitations section. |
| Recommendation: The authors should rerun the CFA using robust estimation methods (e.g., MLR or WLSMV), report full factor matrices, and include additional validity metrics such as AVE and HTMT. A clearer theoretical justification for model modifications and the second-order structure is needed | We would like to thank the reviewer for emphasizing the importance of measurement invariance. Due to sample size imbalances in certain subgroups (particularly regarding gender), conducting a full multi-group invariance test was statistically unfeasible. Consequently, we were unable to include the suggested analysis in our study |
| Conceptual and Reporting Issues:  There are several inconsistencies and gaps in the conceptual framing and reporting. The manuscript alternates between referring to participants as “consumers” and “practitioners,” which creates ambiguity. The term “purchase motivations” is also unclear in the context of yoga participation and should be clarified. | In sports marketing, sports consumers are categorized into two groups: participants (active) and spectators (passive). Those who perform or practice any sports/exercise activity are defined as sports participants. Based on this framework, we have used the terms "consumer" and "participant" interchangeably for yoga practitioners where appropriate. We believe it is conceptually sound to evaluate yoga participants as a type of consumer, similar to general sports participants. |
| The literature review relies heavily on non-academic or gray sources, particularly when citing market size and participation statistics. These claims should be supported with peer-reviewed or authoritative industry data. Additionally, the omission of spirituality and health-related motivations—central themes in yoga literature is not sufficiently justified. Ethical approval is referenced, but the name of the approving board is omitted. Data availability is also vaguely described. The discussion section tends to overstate the novelty of the “Visibility” dimension without sufficient comparative evidence from other cultural contexts. | We acknowledge this limitation. To the best of our knowledge, no reports or datasets on yoga published by official institutions in Türkiye were available at the time of the study. Therefore, we relied on the existing national and international literature and incorporated all relevant and accessible evidence to support the development of the research framework and measurement instrument. (line 555-561) |
| Recommendation: Strengthen the literature review with high-quality sources, clarify terminology, and provide full transparency regarding ethics and data availability. | Bolu Abant Izzet Baysal University has been added (Line 216) |
| Including an appendix with the full scale (in both Turkish and English), scoring instructions, and normative data would improve usability and reproducibility. | Thank you for your valuable feedback. To improve the completeness and clarity of the manuscript, we have included the final renumbered 24-item version of the scale in Tables 8 and 9 at the end of the Results section. These tables present the renumbered items, their corresponding subdimensions, and the minimum and maximum possible scores for each subdimension. We believe that these additions provide a clearer presentation of the scale's structure and scoring procedure. Furthermore, the final 24-item version of the scale in both Turkish and English has been included in the revised manuscript as **Appendix 1**. (Line 410-416) |
| Minor Concerns  There are several minor issues related to language and formatting, including typographical errors, inconsistent spelling (e.g., “Turkey” vs. “Türkiye”), and incomplete figure/table labeling. | The word "Turkey" appears in only two sources in the sources section. One is a book and the other is a ministry report. Both sources are originally written in Turkish, and their English titles are not included in the original texts. Therefore, the authors used the original versions of the works. Apart from this, the word "Turkey" does not appear in the main text. |
| Some tables (e.g., the full EFA matrix) are missing or only partially reported. Clarification of reverse-coded items would also be helpful. | Thank you for your valuable feedback. The scale does not contain any reverse-coded items; therefore, a reverse scoring procedure was not required. |

Reviwer 2

| Comments and Suggestions | Responses |
| --- | --- |
| Severe gender/experience sampling bias limits generalizability • Missing standard analyses: CR/AVE, CMB testing, test-retest reliability | We thank the reviewer for this valuable comment. Test–retest reliability was not assessed in the present study, and this issue has been explicitly acknowledged as a limitation in the manuscript.  Regarding convergent validity indicators, AVE and CR values were examined during the validation process. However, the obtained values did not fully meet the commonly recommended threshold criteria. Therefore, these findings were not considered sufficient evidence of convergent validity. Nevertheless, several other validity and reliability assessments, including exploratory and confirmatory factor analyses, internal consistency coefficients, and McDonald’s omega, were conducted and are fully reported in the manuscript. These analyses generally supported the psychometric adequacy of the proposed measurement instrument. |
| High attrition unexplained; CFA extreme value removal needs thresholds | The following explanations have been added to the conclusion section:  One of the limitations of the present study is that the sample predominantly consisted of Turkish women and individuals who were novice yoga practitioners. This may limit the generalizability of the findings to other demographic groups and practitioners with different levels of yoga experience. Accordingly, this should also be considered an important limitation in the Turkish context. Future studies are therefore encouraged to include more balanced and heterogeneous samples in terms of gender and yoga experience, which may enhance the generalizability and external validity of the findings.  We would like to thank the reviewer for emphasizing the importance of measurement invariance. Due to sample size imbalances in certain subgroups (particularly regarding gender), conducting a full multi-group invariance test was statistically unfeasible. Consequently, we were unable to include the suggested analysis in our study. |
| Required Revisions:  • Address severe sampling bias: 94.9% female (EFA), 89.3% female (CFA), beginner dominant | The following explanations have been added to the conclusion section:  One of the limitations of the present study is that the sample predominantly consisted of Turkish women and individuals who were novice yoga practitioners. This may limit the generalizability of the findings to other demographic groups and practitioners with different levels of yoga experience. Accordingly, this should also be considered an important limitation in the Turkish context. Future studies are therefore encouraged to include more balanced and heterogeneous samples in terms of gender and yoga experience, which may enhance the generalizability and external validity of the findings. |
| • Add missing psychometrics: CR, AVE, omega coefficients, test-retest reliability | We thank the reviewer for this valuable comment. Test–retest reliability was not assessed in the present study, and this issue has been explicitly acknowledged as a limitation in the manuscript.  Regarding convergent validity indicators, AVE and CR values were examined during the validation process. However, the obtained values did not fully meet the commonly recommended threshold criteria. Therefore, these findings were not considered sufficient evidence of convergent validity. Nevertheless, several other validity and reliability assessments, including exploratory and confirmatory factor analyses, internal consistency coefficients, and McDonald’s omega, were conducted and are fully reported in the manuscript. These analyses generally supported the psychometric adequacy of the proposed measurement instrument. |
| Include common method bias testing (Harman's single factor test) | We would like to thank the reviewer for emphasizing the importance of measurement invariance. Due to sample size imbalances in certain subgroups (particularly regarding gender), conducting a full multi-group invariance test was statistically unfeasible. Consequently, we were unable to include the suggested analysis in our study.  Omega coefficients were calculated and reported in the revised manuscript to provide additional evidence regarding the internal consistency reliability of the scale. We acknowledge that test-retest reliability analysis was not conducted in the present study, and this limitation has been explicitly stated in the Limitations section. We agree that future studies should examine the temporal stability of the scale by assessing test-retest reliability. |
| • Explain high EFA attrition (667→429) beyond "open-ended questions" • Provide exact data repository link per PLOS ONE policy | As part of the data preparation process, all responses were subjected to rigorous screening procedures. Cases with missing data, inconsistent response patterns, or other indications of poor data quality were excluded from the analyses. Consequently, the final dataset consisted only of valid and complete responses, thereby improving the reliability of the statistical results.  DOI: 10.5061/dryad.dv41ns2db |
| • Report factor correlation matrix, item descriptives per factor • Justify CFA modification indices (error covariances by content similarity) | The following explanations have been added to the findings section:  The covariance paths between Items 22 and 25 and between Items 15 and 19 were incorporated into the measurement model and are presented in Figure 5. Items 22 and 25 belong to the **Visibility** dimension, whereas Items 15 and 19 belong to the **Socialization** dimension. Within each dimension, these item pairs reflect conceptually similar aspects of the same latent construct. Therefore, the error terms of these items were allowed to covary, as they represent theoretically related content within their respective dimensions |

**Appendix 1**: English and Turkish Final version of the scale

|  | English | Turkish | 1 | 2 | 3 | 4 | 5 |
| --- | --- | --- | --- | --- | --- | --- | --- |
| 1 | I do yoga to escape from the city crowds. | Şehrin kalabalığından kaçmak için yoga yapıyorum. |  |  |  |  |  |
| 2 | I do yoga to meet with new people | Yeni insanlarla tanışmak için yoga yapıyorum. |  |  |  |  |  |
| 3 | I do yoga because I really like its clothes | Kıyafetlerini çok beğendiğim için yoga yapıyorum. |  |  |  |  |  |
| 4 | I do yoga because my doctor referred | Doktorum tavsiye ettiği için yoga yapıyorum. |  |  |  |  |  |
| 5 | I do yoga so that those around me can see that I am a pioneer in yoga | Etrafımdakiler yogada öncü olduğumu görsünler diye yoga yapıyorum. |  |  |  |  |  |
| 6 | I do yoga to escape my financial problems | Ekonomik sorunlarımdan kaçmak için yoga yapıyorum. |  |  |  |  |  |
| 7 | I do yoga to build a social circle | Sosyal çevre edinmek için yoga yapıyorum. |  |  |  |  |  |
| 8 | I do yoga because it gives me self-confidence | Özgüven sağladığı için yoga yapıyorum. |  |  |  |  |  |
| 9 | I do yoga to share on social media | Sosyal medyada paylaşmak için yoga yapıyorum. |  |  |  |  |  |
| 10 | I do yoga to get rid of my unhappiness | Mutsuzluğumu gidermek için yoga yapıyorum. |  |  |  |  |  |
| 11 | I do yoga because my psychotherapist recommended it | Psikoterapistim tavsiye ettiği için yoga yapıyorum. |  |  |  |  |  |
| 12 | I do yoga to escape problems at work | İş yerindeki sorunlardan kaçmak için yoga yapıyorum. |  |  |  |  |  |
| 13 | I do yoga to show off to others | Başkalarına gösteris yapmak için yoga yapıyorum |  |  |  |  |  |
| 14 | I do yoga to be with people who feel in the same way | Aynı şeyleri hisseden insanlar ile birlikte olmak için yoga yapıyorum. |  |  |  |  |  |
| 15 | I do yoga to satisfy my desire to achieve something | Herhangi bir şeyi başarma duygumu tatmin etmek için yoga yapıyorum. |  |  |  |  |  |
| 16 | I do yoga because it's trendy | Moda olduğu için yoga yapıyorum. |  |  |  |  |  |
| 17 | I do yoga to escape environmental issues | Çevresel sorunlardan kaçmak için yoga yapıyorum. |  |  |  |  |  |
| 18 | I do yoga to be around people who do yoga | Yoga yapan insanlarla bir arada olabilmek için yoga yapıyorum. |  |  |  |  |  |
| 19 | I do yoga to get more likes on social media | Sosyal medyada daha fazla beğenilmek için yoga yapıyorum. |  |  |  |  |  |
| 20 | I do yoga because my physical therapist recommended it | Fizyoterapistim tavsiye ettiği için yoga yapıyorum. |  |  |  |  |  |
| 21 | I do yoga to attract other people's attention | Başka insanların ilgisini çekmek için yoga yapıyorum. |  |  |  |  |  |
| 22 | I do yoga to escape political issues | Siyasi sorunlardan kaçmak için yoga yapıyorum. |  |  |  |  |  |
| 23 | I practice yoga because of my ambition to achieve yoga poses | Yoga pozlarını başarmaya yönelik hırsım yüzünden yoga yapıyorum. |  |  |  |  |  |
| 24 | I do yoga to get more followers on social media | Sosyal medyada daha fazla takip edilmek için yoga yapıyorum. |  |  |  |  |  |
