# Supplementary material for: Turkish yoga consumers’ motivation: A scale development study
Source: PLoS One. 2026 Oct 9;21(10):e0359209. doi: 10.1371/journal.pone.0359209 (PMC13652277; doi:10.1371/journal.pone.0359209)
Supplement: Peer review history

# Author comment: Author response to Decision Letter 0

**DOI:** 10.1371/journal.pone.0359209.r001

7 Apr 2026

Attachment Submitted filename: Response to Reviewers.docx

# Peer review: Decision Letter 0

**DOI:** 10.1371/journal.pone.0359209.r002

**Authors:** Santhi Silambanan

11 May 2026

Dear Dr. Eskicioğlu,

Thank you for submitting your manuscript to PLOS ONE. After careful consideration, we feel that it has merit but does not fully meet PLOS ONE’s publication criteria as it currently stands. Therefore, we invite you to submit a revised version of the manuscript that addresses the points raised during the review process.

Please submit your revised manuscript by Jun 25 2026 11:59PM. If you will need more time than this to complete your revisions, please reply to this message or contact the journal office at plosone@plos.org. When you're ready to submit your revision, log on to https://www.editorialmanager.com/pone/ and select the 'Submissions Needing Revision' folder to locate your manuscript file.

- A letter that responds to each point raised by the academic editor and reviewer(s). You should upload this letter as a separate file labeled 'Response to Reviewers'.
- A marked-up copy of your manuscript that highlights changes made to the original version. You should upload this as a separate file labeled 'Revised Manuscript with Track Changes'.
- An unmarked version of your revised paper without tracked changes. You should upload this as a separate file labeled 'Manuscript'.

If you would like to make changes to your financial disclosure, please include your updated statement in your cover letter. Guidelines for resubmitting your figure files are available below the reviewer comments at the end of this letter.

If applicable, we recommend that you deposit your laboratory protocols in protocols.io to enhance the reproducibility of your results. Protocols.io assigns your protocol its own identifier (DOI) so that it can be cited independently in the future. For instructions see: https://journals.plos.org/plosone/s/submission-guidelines#loc-laboratory-protocols. Additionally, PLOS ONE offers an option for publishing peer-reviewed Lab Protocol articles, which describe protocols hosted on protocols.io. Read more information on sharing protocols at https://plos.org/protocols?utm_medium=editorial-email&utm_source=authorletters&utm_campaign=protocols.

As the corresponding author, your ORCID iD is verified in the submission system and will appear in the published article. PLOS supports the use of ORCID, and we encourage all coauthors to register for an ORCID iD and use it as well. Please encourage your coauthors to verify their ORCID iD within the submission system before final acceptance, as unverified ORCID iDs will not appear in the published article. *Only*  the individual author can complete the verification step; PLOS staff *cannot*  verify ORCID iDs on behalf of authors.

We look forward to receiving your revised manuscript.

Kind regards,

Santhi Silambanan, MD, DNB

Academic Editor

PLOS One

Journal Requirements:

When submitting your revision, we need you to address these additional requirements.

1. Please ensure that your manuscript meets PLOS ONE's style requirements, including those for file naming. The PLOS ONE style templates can be found at

https://journals.plos.org/plosone/s/file?id=wjVg/PLOSOne_formatting_sample_main_body.pdf and

https://journals.plos.org/plosone/s/file?id=ba62/PLOSOne_formatting_sample_title_authors_affiliations.pdf

2. Please ensure that you include a title page within your main document. We do appreciate that you have a title page document uploaded as a separate file, however, as per our author guidelines (http://journals.plos.org/plosone/s/submission-guidelines#loc-title-page) we do require this to be part of the manuscript file itself and not uploaded separately.

Could you therefore please include the title page into the beginning of your manuscript file itself, listing all authors and affiliations.

3. Please ensure that you include a title page within your main document. You should list all authors and all affiliations as per our author instructions and clearly indicate the corresponding author.

4. Please include a separate caption for each figure in your manuscript

5. When completing the data availability statement of the submission form, you indicated that you will make your data available on acceptance. We strongly recommend all authors decide on a data sharing plan before acceptance, as the process can be lengthy and hold up publication timelines. Please note that, though access restrictions are acceptable now, your entire data will need to be made freely accessible if your manuscript is accepted for publication. This policy applies to all data except where public deposition would breach compliance with the protocol approved by your research ethics board. If you are unable to adhere to our open data policy, please kindly revise your statement to explain your reasoning and we will seek the editor's input on an exemption. Please be assured that, once you have provided your new statement, the assessment of your exemption will not hold up the peer review process.

If the reviewer comments include a recommendation to cite specific previously published works, please review and evaluate these publications to determine whether they are relevant and should be cited. There is no requirement to cite these works unless the editor has indicated otherwise.

Additional Editor Comments:

The authors shall respond to the queries raised by the reviewers.

[Note: HTML markup is below. Please do not edit.]

Reviewers' comments:

Reviewer's Responses to Questions

**Comments to the Author**

1. Is the manuscript technically sound, and do the data support the conclusions?

Reviewer #1: Partly

Reviewer #2: Yes

**********

2. Has the statistical analysis been performed appropriately and rigorously?

Reviewer #1: Yes

Reviewer #2: Yes

**********

3. Have the authors made all data underlying the findings in their manuscript fully available?

The PLOS Data policy

Reviewer #1: No

Reviewer #2: Yes

**********

4. Is the manuscript presented in an intelligible fashion and written in standard English?

Reviewer #1: Yes

Reviewer #2: Yes

**********

Reviewer #1: The manuscript presents a rigorous and well-structured scale development study for the Yoga Motivation Scale (YMS), tailored to Turkish yoga practitioners. It identifies five dimensions: Visibility (8 items), Escape (6 items), Socialization (4 items), Reference (3 items), and Achievement (4 items) and follows a generally sound methodological progression from item generation to validation.

The authors move systematically from an initial pool of 118 items to 51 items using content validity (Lawshe CVR = 0.75 with 9 experts), followed by exploratory factor analysis (EFA; n = 429, KMO = 0.871, 62.7% explained variance, loadings > 0.60) and confirmatory factor analysis (CFA; n = 224, RMSEA = 0.077–0.079, CFI = 0.926–0.930 after modifications). Overall, the study reflects a solid application of established scale development practices. However, several substantive issues need to be addressed before the manuscript is suitable for publication.

Strengths:

The study offers a clear and timely rationale for developing a new motivation scale in an emerging yoga market, especially distinguishing it from traditional exercise motivation instruments such as EMI-2. The three-phase scale development process aligns well with established methodological guidelines, and the use of separate samples for EFA and CFA strengthens the validity of the findings.

Reliability metrics are strong, with the overall scale demonstrating high internal consistency (α = 0.90, ω = 0.89), and most sub-dimensions also performing well (α = 0.63–0.96). Notably, the introduction of the “Visibility” dimension capturing social media presence and aesthetic motivations is both novel and contextually relevant, particularly given the COVID-era data collection period and the influence of Instagram culture in Turkey.

Major Concerns:

Sample and Generalizability-

The sample composition raises concerns about the generalizability of the findings. There is a strong gender imbalance (EFA: 94.9% female; CFA: 89.3% female), along with a dominance of relatively inexperienced practitioners (over 57% with less than three years of experience in the EFA sample). This limits the applicability of the scale across broader yoga populations.

Additionally, the use of convenience sampling through yoga centers may introduce self-selection bias, potentially overrepresenting individuals motivated by social or visibility-related factors. The manuscript does not include subgroup analyses (e.g., by gender or experience level), nor does it assess measurement invariance. Response rates and potential non-response bias are also not adequately addressed, particularly given the substantial amount of missing demographic data.

Recommendation:

The authors should aim to recruit a more balanced sample, ideally stratified by gender and experience level. At minimum, they should limit their claims to female and novice Turkish yoga practitioners. Reporting response flow, addressing missing data, and conducting invariance testing would significantly strengthen the study.

Psychometric Analysis:

While the factor analyses are generally well executed, several gaps remain. In the EFA, key diagnostics such as parallel analysis and scree plot interpretation are underreported. Extremely high factor loadings (up to 0.96) raise concerns about potential redundancy or suppression effects, and the manuscript does not provide full item-level statistics (e.g., communalities, uniqueness).

In the CFA, the use of maximum likelihood estimation assumes normality, but tests for this assumption are not sufficiently reported. Model modifications (e.g., correlated error terms) are introduced without clear theoretical justification. The study also lacks additional validity checks, including convergent and discriminant validity (e.g., AVE, CR, HTMT), and does not address common method bias (e.g., Harman’s single-factor test).

Furthermore, the second-order factor model is mentioned but not adequately explained, particularly in terms of factor loadings and theoretical interpretation. The absence of test–retest reliability and criterion validity (e.g., comparison with existing scales) also limits the robustness of the validation.

Recommendation:

The authors should rerun the CFA using robust estimation methods (e.g., MLR or WLSMV), report full factor matrices, and include additional validity metrics such as AVE and HTMT. A clearer theoretical justification for model modifications and the second-order structure is needed.

Conceptual and Reporting Issues:

There are several inconsistencies and gaps in the conceptual framing and reporting. The manuscript alternates between referring to participants as “consumers” and “practitioners,” which creates ambiguity. The term “purchase motivations” is also unclear in the context of yoga participation and should be clarified.

The literature review relies heavily on non-academic or gray sources, particularly when citing market size and participation statistics. These claims should be supported with peer-reviewed or authoritative industry data. Additionally, the omission of spirituality and health-related motivations—central themes in yoga literature is not sufficiently justified.

Ethical approval is referenced, but the name of the approving board is omitted. Data availability is also vaguely described. The discussion section tends to overstate the novelty of the “Visibility” dimension without sufficient comparative evidence from other cultural contexts.

Recommendation:

Strengthen the literature review with high-quality sources, clarify terminology, and provide full transparency regarding ethics and data availability. Including an appendix with the full scale (in both Turkish and English), scoring instructions, and normative data would improve usability and reproducibility.

Minor Concerns

There are several minor issues related to language and formatting, including typographical errors, inconsistent spelling (e.g., “Turkey” vs. “Türkiye”), and incomplete figure/table labeling. Some tables (e.g., the full EFA matrix) are missing or only partially reported. Clarification of reverse-coded items would also be helpful.

Overall Recommendation

Accept with Major Revisions.

The manuscript presents a promising and contextually relevant scale with a solid methodological foundation. However, important concerns related to sampling bias, psychometric rigor, and reporting clarity must be addressed to meet publication standards. With these revisions, the study has the potential to make a valuable contribution to the literature on yoga motivation and consumer behavior.

Reviewer #2: The manuscript demonstrates solid scale development methodology following DeVellis (2014) and Boateng et al. (2018), successfully creating a 24-item, 5-factor Yoga Motivation Scale (Visibility, Escape, Socialization, Reference, Achievement) with acceptable EFA (KMO=0.871, 62.7% variance) and CFA fit (χ²/df=2.32, CFI=0.93, RMSEA=0.077). The novel "Visibility" factor capturing social media/fashion influences is particularly relevant for emerging markets like Turkey.

Justification needed for the following statements:

• Addresses clear gap: first yoga motivation scale for Turkey/emerging markets

• Psychometric rigor suitable for PLOS ONE (separate EFA/CFA samples)

• Novel theoretical contribution: "Visibility" dimension for consumer behavior.

Key Concerns for Authors:

• Severe gender/experience sampling bias limits generalizability

• Missing standard analyses: CR/AVE, CMB testing, test-retest reliability

• High attrition unexplained; CFA extreme value removal needs thresholds

**********

what does this mean?). If published, this will include your full peer review and any attached files.

If you choose “no”, your identity will remain anonymous but your review may still be made public.

**Do you want your identity to be public for this peer review?**  For information about this choice, including consent withdrawal, please see our Privacy Policy

Reviewer #1: **Yes:**  Sai Charan Reddy Bhimavarapu

Reviewer #2: **Yes:**  Dr D Elanchezhiyan, Research Officer, CRIYN, CCRYN, New Delhi, India

**********

[NOTE: If reviewer comments were submitted as an attachment file, they will be attached to this email and accessible via the submission site. Please log into your account, locate the manuscript record, and check for the action link "View Attachments". If this link does not appear, there are no attachment files.]

To ensure your figures meet our technical requirements, please review our figure guidelines: https://journals.plos.org/plosone/s/figures

You may also use PLOS’s free figure tool, NAAS, to help you prepare publication quality figures: https://journals.plos.org/plosone/s/figures#loc-tools-for-figure-preparation.

NAAS will assess whether your figures meet our technical requirements by comparing each figure against our figure specifications.

# Author comment: Author response to Decision Letter 1

**DOI:** 10.1371/journal.pone.0359209.r003

27 Jun 2026

The following explanations have been added to the conclusion section:

One of the limitations of the present study is that the sample predominantly consisted of Turkish women and individuals who were novice yoga practitioners. This may limit the generalizability of the findings to other demographic groups and practitioners with different levels of yoga experience. Accordingly, this should also be considered an important limitation in the Turkish context. Future studies are therefore encouraged to include more balanced and heterogeneous samples in terms of gender and yoga experience, which may enhance the generalizability and external validity of the findings.

We thank the reviewer for this valuable comment. We acknowledge the limitation associated with the use of convenience sampling. However, data collection for the EFA phase was carried out during the COVID-19 pandemic, when government-imposed restrictions substantially constrained access to participants. Therefore, convenience sampling was adopted as the most practical data collection method. To enhance data quality, all responses were carefully screened, and incomplete or inconsistent cases were excluded from the analyses.

Given the relatively high factor loadings (up to .96) observed for some items, we carefully reexamined the item pool for potential item redundancy and suppression effects. The results indicated that these items were theoretically and conceptually consistent with their respective latent constructs. Therefore, the high factor loadings were interpreted as reflecting a strong relationship between the items and the underlying constructs.

The following explanations have been added to the findings section:

The covariance paths between Items 22 and 25 and between Items 15 and 19 were incorporated into the measurement model and are presented in Figure 5. Items 22 and 25 belong to the Visibility dimension, whereas Items 15 and 19 belong to the Socialization dimension. Within each dimension, these item pairs reflect conceptually similar aspects of the same latent construct. Therefore, the error terms of these items were allowed to covary, as they represent theoretically related content within their respective dimensions. (Line 361-367)

We would like to thank the reviewer for emphasizing the importance of measurement invariance. Due to sample size imbalances in certain subgroups (particularly regarding gender), conducting a full multi-group invariance test was statistically unfeasible. Consequently, we were unable to include the suggested analysis in our study.

Thank you for this valuable comment. The absence of test-retest reliability and criterion validity analyses had already been acknowledged in the Limitations section.

We would like to thank the reviewer for emphasizing the importance of measurement invariance. Due to sample size imbalances in certain subgroups (particularly regarding gender), conducting a full multi-group invariance test was statistically unfeasible. Consequently, we were unable to include the suggested analysis in our study

In sports marketing, sports consumers are categorized into two groups: participants (active) and spectators (passive). Those who perform or practice any sports/exercise activity are defined as sports participants. Based on this framework, we have used the terms "consumer" and "participant" interchangeably for yoga practitioners where appropriate. We believe it is conceptually sound to evaluate yoga participants as a type of consumer, similar to general sports participants.

We acknowledge this limitation. To the best of our knowledge, no reports or datasets on yoga published by official institutions in Türkiye were available at the time of the study. Therefore, we relied on the existing national and international literature and incorporated all relevant and accessible evidence to support the development of the research framework and measurement instrument. (line 555-561)

Bolu Abant Izzet Baysal University has been added (Line 216)

Thank you for your valuable feedback. To improve the completeness and clarity of the manuscript, we have included the final renumbered 24-item version of the scale in Tables 8 and 9 at the end of the Results section. These tables present the renumbered items, their corresponding subdimensions, and the minimum and maximum possible scores for each subdimension. We believe that these additions provide a clearer presentation of the scale's structure and scoring procedure. Furthermore, the final 24-item version of the scale in both Turkish and English has been included in the revised manuscript as Appendix 1. (Line 410-416)

The word "Turkey" appears in only two sources in the sources section. One is a book and the other is a ministry report. Both sources are originally written in Turkish, and their English titles are not included in the original texts. Therefore, the authors used the original versions of the works. Apart from this, the word "Turkey" does not appear in the main text.

Thank you for your valuable feedback. The scale does not contain any reverse-coded items; therefore, a reverse scoring procedure was not required.

Attachment Submitted filename: Responses.docx

# Peer review: Decision Letter 1

**DOI:** 10.1371/journal.pone.0359209.r004

**Authors:** Santhi Silambanan

10 Sep 2026

TURKISH YOGA CONSUMERS' MOTIVATION: A SCALE DEVELOPMENT STUDY

PONE-D-26-13529R1

Dear Dr. Eskicioğlu,

We’re pleased to inform you that your manuscript has been judged scientifically suitable for publication and will be formally accepted for publication once it meets all outstanding technical requirements.

Within one week, you’ll receive an e-mail detailing the required amendments. When these have been addressed, you’ll receive a formal acceptance letter and your manuscript will be scheduled for publication.

An invoice will be generated when your article is formally accepted. Please note, if your institution has a publishing partnership with PLOS and your article meets the relevant criteria, all or part of your publication costs will be covered. Please make sure your user information is up-to-date by logging into Editorial Manager at Editorial Manager® and clicking the ‘Update My Information' link at the top of the page. For questions related to billing, please contact billing support.

If your institution or institutions have a press office, please notify them about your upcoming paper to help maximize its impact. If they’ll be preparing press materials, please inform our press team as soon as possible -- no later than 48 hours after receiving the formal acceptance. Your manuscript will remain under strict press embargo until 2 pm Eastern Time on the date of publication. For more information, please contact onepress@plos.org.

Kind regards,

Santhi Silambanan, MD, DNB

Academic Editor

PLOS One

# Editor report: Acceptance letter

**DOI:** 10.1371/journal.pone.0359209.r005

**Authors:** Santhi Silambanan

PONE-D-26-13529R1

PLOS One

Dear Dr. Eroglu Eskicioglu,

I'm pleased to inform you that your manuscript has been deemed suitable for publication in PLOS One. Congratulations! Your manuscript is now being handed over to our production team.

At this stage, our production department will prepare your paper for publication. This includes ensuring the following:

* All references, tables, and figures are properly cited

* All relevant supporting information is included in the manuscript submission,

* There are no issues that prevent the paper from being properly typeset

You will receive further instructions from the production team, including instructions on how to review your proof when it is ready. Please keep in mind that we are working through a large volume of accepted articles, so please give us a few days to review your paper and let you know the next and final steps.

Lastly, if your institution or institutions have a press office, please let them know about your upcoming paper now to help maximize its impact. If they'll be preparing press materials, please inform our press team within the next 48 hours. Your manuscript will remain under strict press embargo until 2 pm Eastern Time on the date of publication. For more information, please contact onepress@plos.org.

You will receive an invoice from PLOS for your publication fee after your manuscript has reached the completed accept phase. If you receive an email requesting payment before acceptance or for any other service, this may be a phishing scheme. Learn how to identify phishing emails and protect your accounts at https://explore.plos.org/phishing.

If we can help with anything else, please email us at customercare@plos.org.

Thank you for submitting your work to PLOS One and supporting open access.

Kind regards,

PLOS One Editorial Office Staff

on behalf of

Dr. Santhi Silambanan

Academic Editor

PLOS One
